# Exploring personalized neoadjuvant therapy selection strategies in breast cancer: an explainable multi-modal response model

**DOI:** 10.1016/j.eclinm.2025.103356

**Published:** 2025-07-17

**Authors:** Luyi Han, Tianyu Zhang, Anna D'Angelo, Anna van der Voort, Katja Pinker-Domenig, Marleen Kok, Gabe Sonke, Yuan Gao, Xin Wang, Chunyao Lu, Xinglong Liang, Jonas Teuwen, Tao Tan, Ritse Mann

**Affiliations:** a Department of Radiology and Nuclear Medicine, Radboud University Medical Centre, Geert Grooteplein 10, Nijmegen, 6525 GA, the Netherlands; b Department of Radiology, The Netherlands Cancer Institute, Plesmanlaan 121, Amsterdam, 1066 CX, the Netherlands; c Faculty of Applied Sciences, Macao Polytechnic University, 999078, Macao Special Administrative Region of China; d GROW School for Oncology and Developmental Biology, Maastricht University Medical Centre, P. Debyelaan 25, Maastricht, 6202 AZ, the Netherlands; e Dipartimento di diagnostica per immagini, Radioterapia, Oncologia ed ematologia, Fondazione Universitaria A. Gemelli, IRCCS Roma, Roma, Italy; f Department of Internal Medicine, Dijklander Hospital, Hoorn, the Netherlands; g Division of Breast Imaging, Department of Radiology, Columbia University, Vagelos College of Physicians and Surgeons, New York, USA; h Department of Medical Oncology, The Netherlands Cancer Institute, Plesmanlaan 121, Amsterdam, 1066 CX, the Netherlands; i Department of Radiation Oncology, The Netherlands Cancer Institute, Plesmanlaan 121, Amsterdam, 1066 CX, the Netherlands

**Keywords:** Breast cancer, Neoadjuvant therapy, Precise medicine, Multi-modal learning, Explainable artificial intelligence

## Abstract

**Background:**

Neoadjuvant therapy (NAT) regimens for breast cancer are generally determined according to cancer stage and molecular subtypes without fully considering the inter-patient variability, which may lead to inefficiency or overtreatment. Artificial intelligence (AI) may support personalized regimen recommendations by learning the synergistic relationship between pre-NAT individual-patient data, regimens, and corresponding short- or long-term therapy responses.

**Methods:**

In this retrospective study, we collected data from breast cancer patients treated with NAT between 2000 and 2020 from the Netherlands and the USA. Median follow-up times ranged from 3·7 to 4·9 years across molecular subtypes and cohorts. We developed and externally validated a multi-modal model integrating pre-NAT clinical data, dynamic contrast enhanced (DCE)-MRI images, and medical reports to predict pathological complete response (pCR) and likelihood of survival after NAT. We subsequently evaluated potential benefits for patients receiving a personalized regimen recommended based on these predictions.

**Findings:**

We trained our model on 655 patients and validated it on internal (655 patients) and external (241 patients) cohorts. Given the factual regimens, the model can correctly predict the corresponding therapy response, with areas under the receiver operating characteristic curves (AUC) of 0·80 (95% CI 0·73–0·87), 0·75 (0·66–0·83), and 0·85 (0·77–0·92) for pCR prediction of human epidermal growth factor receptor 2 (HER2)+, triple-negative, and estrogen receptor/progesterone receptor (ER/PR)+&HER2− patients in the internal validation cohort, respectively. Performance in the external validation cohort was 0·707 (0·557–0·836), 0·558 (0·359–0·749), and 0·860 (0·767–0·945) for the corresponding molecular subtypes, respectively. In the internal validation cohort, survival prediction identified high-risk patients across different molecular subtypes, as demonstrated by a hazard ratio (HR) of 3·29 (0·91–11·94) (HER2+), 3·54 (1·52–8·20) (triple-negative), and 2·78 (1·45–5·31) (ER/PR+&HER2−), albeit results were not significant for HER2+ cancers.

**Interpretation:**

Our findings indicate that the prognostic scores generated by the response model could identify patient subgroups with relatively poor outcomes under their actual treatments. These preliminary findings may inform future efforts toward personalized NAT regimen selection beyond traditional criteria such as cancer stage and subtype, but should be interpreted cautiously and validated in prospective studies with longer follow-up because these tumors can relapse at a later stage.

**Funding:**

None.

**Research in context**

**Evidence before this study**

We searched in PubMed on March 1, 2024, without language or date restrictions from database inception, using the terms: “neoadjuvant” AND “breast cancer” AND (“prediction” OR “response” OR “personalized” OR “recommendation” OR “regimen”) AND (“deep learning” OR “artificial intelligence”). We identified 79 original studies that applied artificial intelligence to breast cancer NAT response prediction, where 29 studies utilized deep learning features or radiomics derived from dynamic contrast-enhanced (DCE)-MRI, 18 relied on ultrasound imaging, 20 focused on histopathology images, and eight incorporated other data modalities. Only two studies employed multimodal data integration, and only two attempted to predict survival outcomes post-NAT, but did not account for variations in NAT regimens among patients or evaluate the impact of regimen selection on treatment outcomes.

**Added value of this study**

In this study, we developed a multi-modal model that integrates deep learning and multi-modal data to provide personalized NAT recommendations for breast cancer patients. This model leverages pre-treatment clinical data, DCE-MRI imaging, and medical records to predict the likelihood of pCR and assign individualized risk scores, which may facilitate tailored regimen recommendations.

**Implications of all the available evidence**

Our multi-modal response model may offer pre-treatment prognostic assessment by considering individual patient variability and expected response. If validated in a prospective cohort with longer follow-up, this approach could potentially inform personalized NAT regimen recommendations for breast cancer patients.

## Introduction

Neoadjuvant therapy (NAT) is a standard treatment for locally advanced breast cancer^1^ and nowadays is increasingly used for smaller tumors with aggressive biological characteristics.^2^ NAT can facilitate breast-conserving and axillary sparing surgery by reducing tumor burden.^2^ Achieving pathological complete response (pCR) after NAT is associated with favorable long-term outcomes, allowing guidance of further treatment.^3^, ^4^, ^5^ The response to NAT varies across molecular subtypes of breast cancer, primarily defined by estrogen receptor/progesterone receptor (ER/PR) and human epidermal growth factor receptor 2 (HER2) status, with triple-negative (HER2−&ER/PR−) and HER2+ tumors showing the highest pCR rates.^6^^,^^7^ According to clinical guidelines,^1^ current NAT mainly involve polychemotherapy with an anthracycline plus taxane backbone (e.g., AC-T) for triple-negative patients, chemotherapy combined with HER2 antibodies (specifically trastuzumab and pertuzumab) for HER2+ tumors, and neoadjuvant chemotherapy or endocrine therapy (e.g., tamoxifen) for ER/PR+ breast cancer. However, responses to NAT vary among individuals. Some HER2+ tumors show pCR as early as 14 days after starting treatment (complete treatment may involve up to six cycles), but each additional cycle of treatment increases toxicity.^7^, ^8^, ^9^ Some tumors do not respond to treatment, and the NAT regimen should be adjusted promptly to maximize the benefits of treatment. Therefore, personalized prediction of NAT response to determine whether a patient will achieve pCR or prolong survival can optimize the selection of NAT regimens and avoid inefficiency or overtreatment.

Existing prognostic systems, based on molecular subtypes, TNM staging, genomic risk using gene expression profiling and evolving prognostic markers such as tumor-infiltrating lymphocytes (TILs),^10^ do not consider the heterogeneity observed in radiological imaging and histopathological tissue sections of breast cancer. Imaging biomarkers obtained from MRI measurements, e.g., the functional tumor volume (FTV) derived from dynamic contrast-enhanced (DCE)-MRI and the apparent diffusion coefficient (ADC) derived from diffusion-weighted imaging (DWI), have been found to be associated with the response to NAT.^11^^,^^12^ Unstructured medical reports, including radiology and pathology reports, can also yield high-quality features for prediction.^13^^,^^14^ Recently, deep learning has been extensively utilized to assess NAT response, automatically extracting predictive features across modalities, improving pCR prediction and risk stratification.^15^, ^16^, ^17^, ^18^, ^19^

However, prior studies are limited to single NAT regimens and do not consider counterfactual outcomes of alternative regimens, and further lack of model interpretability.^15^, ^16^, ^17^ The selection bias in non-randomized controlled trials further complicate comparisons of regimen effectiveness.^20^ Previous studies,^21^^,^^22^ such as balanced representation learning, aim to mitigate selection bias by mapping patient characteristics into a latent space where treated and control groups achieve better covariate balance. While being effective in addressing confounding, these approaches are typically applied to tabular data and do not explicitly model uncertainties in counterfactual predictions. Other methods leverage generative models, such as generative adversarial networks (GANs) and variational autoencoders (VAEs), to infer hidden unobserved variables and estimate counterfactual outcome distributions. However, these studies predominantly focus on single-modality data, overlooking the multimodal integration of clinical variables, imaging, and textual reports, which is critical for oncological decision-making. Incorporating multimodal fusion with these representation learning techniques enables a more comprehensive and interpretable approach to treatment outcome estimation.

To bridge these gaps, we developed a multi-modal probabilistic model to predict pCR and survival outcomes for breast cancer patients by integrating multiple AI-driven pre-NAT information, specifically including clinical data, DCE-MRI, and medical reports. By leveraging contrastive language-image pre-training (CLIP),^23^ our model aligns and compresses tabular, image, and report data into low-dimensional features, which helps extract keywords from reports related to image features and allows the model to focus on the image area described by the reports. We further explored the mechanism by which image- and report-based information may correct the clinical data-based predictions, also supporting from treatment-response-related keywords on reports. Additionally, we categorized and recommended patients into three treatment groups based on the predicted scores, recommending lower-toxicity regimens, higher-toxicity regimens, or enrollment in clinical trials, respectively.

## Methods

### Ethics

This retrospective study was approved by the Institutional Review Board of the Netherlands Cancer Institute (NKI), with registration number IRBd21-059. The protocol was not otherwise published. All data were collected and utilized in compliance with local ethical regulations. The requirement to obtain informed consent was waived by the ethics committees due to the exclusive use of anonymized patient-level data.

### Patient cohorts

In this retrospective multicenter study, we used anonymized pre-NAT clinical data, DCE-MRI, and medical reports from cohorts in the Netherlands and the USA. The NKI cohort (Netherlands, n = 2150) included breast cancer patients undergoing NAT at the NKI between 2000 and 2020. 840 patients who lack key clinical data (receptor status and TNM staging), pCR status, follow-up information, or NAT regimen records were excluded. The final cohort consisted of 1310 patients (see Appendix Figure S17), randomly divided into two sets in a 1:1 ratio: 655 patients for five-fold cross-validation training and 655 patients for internal validation. The DUKE cohort (USA, n = 241) served as an external validation cohort, which selected patients who underwent NAT from the Duke-Breast-Cancer-MRI dataset^24^ comprising 922 biopsy-confirmed invasive breast cancer patients. The NKI and DUKE cohorts contain the outcome of NAT response (pCR or no-pCR) and overall survival. We did not observe specific other variations in this population and therefore assumed the missing data is indeed random. Detailed inclusion and exclusion criteria are in Appendix Figure S1.

### Experimental design and statistics

The response model estimated the treatment effects, consisting of patient-level pCR and risk scores, for all potential NAT regimens (anthracycline backbone, anthracycline plus taxane backbone, single HER2 antibody, multiple HER2 antibodies, and hormone therapy). To evaluate the ability of the response model to predict factual outcomes from observed regimens, the areas under the receiver operating characteristic curves (AUC) were calculated to obtain pCR scores, and concordance (C)-indexes were utilized to evaluate risk scores for overall survival. pCR (ypT0/isN0) was defined as the absence of invasive carcinoma within the breast and lymph nodes, regardless of the presence of carcinoma in situ, on the surgical sample after NAT. Overall survival was defined as the duration between surgery and either death from any cause or last follow-up.

**Fig. 1:**
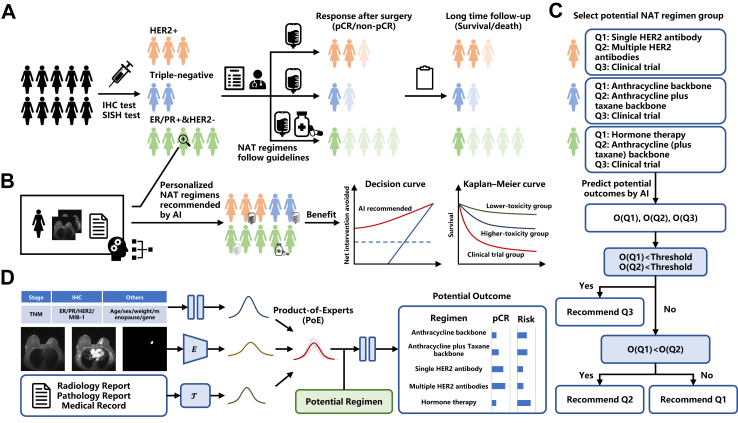
Clinical need and outline of the study. (A) Guideline-based neoadjuvant therapy (NAT) regimen selection and prognostic outcomes for breast cancer patients in clinical practice. (B) Combined with imaging and report data, the deep learning model can offer personalized NAT regimen recommendations and improve patient prognosis. (C) Flowchart of personalized NAT regimen recommendation based on outcome scores predicted from the model. (D) Various prognostic-related posterior distributions were derived from multi-modal data using multiple encoders. These distributions were then combined into a single distribution using the Product-of-Experts (PoE) approach. The combined distribution and potential NAT regimens were utilized to predict prognosis outcomes, including pathological complete response (pCR) and risk scores. IHC: immunohistochemistry. SISH: silver-enhanced in situ hybridization.

We categorized NAT regimens into three groups based on molecular subtypes of breast cancer, with each group containing two to three regimens that comply with guidelines in Fig. 1. Within each group, patients were recommended for a lower-toxicity regimen (Q1 scores were higher than or equal to Q2 scores), a higher-toxicity regimen (Q1 scores were lower than Q2 scores), or participating in a clinical trial with a potentially better working agent, accepting even slightly higher toxicity (both Q1 and Q2 scores were below a threshold). When we focus on the short-term effects of NAT, pCR scores were utilized for regimen recommendation, with the Youden index of pCR scores of the training set as a threshold. Likewise, negative risk scores were used for regimen recommendations focused on long-term effects, with the 25% cutoffs of the negative risk scores of the training set as a threshold. We then investigated whether the deep learning-based NAT regimen recommendation strategy could provide additional prognostic value in patients with breast cancer. Ideally, for patients treated with a lower-toxicity regimen, the model would recommend the same regimen for patients with better outcomes, a higher-toxicity regimen for patients with moderate outcomes, and clinical trials with new active drugs for patients with worse outcomes. For patients treated with a higher-toxicity regimen, the model should also recommend clinical trials for patients with the poorest outcomes, but it could recommend a lower-toxicity regimen for patients with a high likelihood of achieving pCR on such a regimen, even though in the context of this study this cannot be directly validated. Decision curve analysis was used to evaluate the potential benefits, i.e., achieving pCR and avoiding overtreatment, brought by deep learning-based regimen recommendations.

### Data preprocessing and deep-learning procedures

Multi-modal pre-NAT information (clinical data, DCE-MRI, and medical reports) was introduced to predict treatment effects. Clinical data comprised patient information (age, gender, weight, and menopause status), immunohistochemistry (IHC), tumor stage, and presence and type of hereditary genetic mutations. Age and weight were represented using numeric encoding, normalizing to (0, 1). Other clinical data were encoded to vectors. IHC consists of ER, PR, HER2, and MIB-1 status, encoded as 1 for positive and 0 for negative. The threshold of ER and PR positive is set as 1%. In addition, the positive percentage of ER, PR, and MIB-1 is presented as a numeric value of (0, 1), and the HER2 level is categorized as [1, 0, 0] for 0, [0, 1, 0] for 1, and [0, 0, 1] for 3+. As shown in Appendix Figure S2, DCE-MRI preprocessing involved protocol standardization, image normalization, and tumor segmentation. To avoid domain bias within DCE-MRI acquiring from different protocols and inclusion periods, we unify MRI sequences to fat-saturated pre- and post-contrast DCE-MRI with Seq2Seq^25^ and detect breast cancer regions with TSF-Seq2Seq.^26^ To train the response model, all the images and corresponding segmentations were resampled to a voxel size of 2 × 2 × 2 mm, central cropped or padded to the size of 88 × 176 × 176 voxels, and normalized to [0, 1] using 99·5% intensity cutoffs. Appendix Figures S4 and S5 and Table S4 illustrated that the proposed preprocessing protocol can improve pCR and overall survival prediction. Only the NKI cohort had pre-NAT medical reports, including radiology reports (for MRI, ultrasound, mammography, etc.), pathology reports for diagnosis, and patient history medical records. All privacy-related content, such as the patient's name, identification code, and transfer hospital information, has been removed. These reports in Dutch were tokenized and padded to ensure a uniform length of 512 tokens, with blank text substituting for missing data.

We propose a multi-modal probabilistic response model to learn the relationship between individual patient data, observed regimen, and corresponding factual treatment outcome in Fig. 1. Multi-modal information were first encoded into 256-dimensional feature vectors via a multi-layer perception (MLP) for clinical data, a ResNet-based image encoder^25^ for DCE-MRI, and a BERT-based text encoder (RadioLOGIC)^14^ for medical reports. The encoders, pre-trained via CLIP, aligned features into unified spaces. Then, we separately estimated the true posterior distributions of prognosis with the dimension of 3 from the feature vectors of input data, and combined multiple distributions into a single one using the Product-of-Experts (PoE) method.^27^ Finally, the outcomes of pCR and risk scores were predicted using MLP layers from the combination of the given regimen and the sampling of the true posterior distribution.

### Multi-modal response model

The response model is proposed to predict the personalized treatment outcome *Y* based on observed covariates *X* and given regimen *R*. Treatment outcome $Y=\left\{y_{pcr},y_{followup}\right\}$ involves pCR and overall survival prediction. Observed covariates $X=\left\{x_{tab},x_{img},x_{txt}\right\}$ consist of clinical data, MRI images, and medical reports. NAT regimens $R=\left\{r_{1},r_{2},r_{3},r_{4},r_{5}\right\}$ include $r_{1}=\left[1,0,0,0,0\right]$ for anthracycline backbone, $r_{2}=\left[0,1,0,0,0\right]$ for anthracycline plus taxane backbone, $r_{3}=\left[0,0,1,0,0\right]$ for single HER2 antibody, $r_{4}=\left[0,0,0,1,0\right]$ for multiple HER2 antibodies, and $r_{5}=\left[0,0,0,0,1\right]$ for hormone therapy.

As shown in Appendix Figure S3, the response model involves a clinical data encoder $E_{tab}$, an image encoder $E_{img}$, and a text encoder $E_{txt}$. First, we use different encoders to extract the corresponding features, where $f_{tab}=E_{tab}\left(x_{tab}\right)$, $f_{img}=E_{img}\left(x_{img}\right)$, and $f_{txt}=E_{txt}\left(x_{txt}\right)$. We then generate the representation conditioned on different regimens by combining the extracted features with their respective regimen assignments *r* and estimate the mean ($\mu_{tab}$, $\mu_{img}$, $\mu_{txt}$) and covariances ($\sigma_{tab}$, $\sigma_{img}$, $\sigma_{txt}$) for the distribution of each input. Then, we use the Product-of-Experts (PoE) method to combine the multiple distributions into a single one, and the joint distribution is defined as follows,

$$\mu_{PoE}=\left(\frac{\mu_{prior}}{\sigma_{prior}^{2}}+\frac{\mu_{tab}}{\sigma_{tab}^{2}}+\frac{\mu_{img}}{\sigma_{img}^{2}}+\frac{\mu_{txt}}{\sigma_{txt}^{2}}\right)\cdot \sigma_{PoE}^{2}$$

$$\sigma_{PoE}^{2}={\left(\frac{1}{\sigma_{prior}^{2}}+\frac{1}{\sigma_{tab}^{2}}+\frac{1}{\sigma_{img}^{2}}+\frac{1}{\sigma_{txt}^{2}}\right)}^{-1}$$

where $\mu_{prior}$ and $\sigma_{prior}$ represent the mean and covariance of the universal prior expert, which is typically a spherical Gaussian distribution N(0,1).

By reparameterization, we can sample prognostic scores from the estimated prior distribution p(z|x,r) for the prediction of pCR and overall survival. We use prognostic scores of clinical data, MRI images, medical reports, and PoE to predict pCR and overall survival, respectively, and additionally predict a correction factor α, using the PoE results to correct the results predicted by clinical data as follows,

$${\hat{y}}_{correct}=\alpha \;\cdot \;{\hat{y}}_{PoE}\;+\;\left(1-\alpha \right)\;\cdot \;{\hat{y}}_{tab}$$

### Training loss

The evidence lower bound (ELBO) of the response model is given referring to,

$$ELBO=E_{z∼q}\;\log\;p\left(y|z,r\right)\;-\;\lambda_{KL}D_{KL}\left(q\left(z|x,y,r\right)\|p\left(z|x,r\right)\right)$$

where $D_{KL}\left(q\left(z|x,y,r\right)\|p\left(z|x,r\right)\right)$ is the Kullback–Leibler (KL) divergence between q(z|x,y,r) and p(z|x,r). $\lambda_{KL}$ is the balance term in the ELBO and is set to be 1 in this work. The cross-entropy loss was utilized as the loss function for pCR prediction as follows,

$$L_{pcr}=-y_{pcr}\;\log\left({\hat{y}}_{pcr}\right)\;-\;\left(1-y_{pcr}\right)\log\left(1-{\hat{y}}_{pcr}\right)$$

where $y_{pcr}$ is the label of pCR status (0 for non-pCR and 1 for pCR), ${\hat{y}}_{pcr}$ is the pCR score predicted by the response model. The Cox partial likelihood was utilized as the loss function for overall survival prediction as follows,

$$L_{cox}=-\sum_{i:E_{i}=1}\left({\hat{y}}_{followup}\left(x_{i}\right)\;-\;\log\sum_{j:T_{j}>T_{i}}e^{{\hat{y}}_{followup}\left(x_{j}\right)}\right)$$

where $x_{i}$ present the features of the *i* th sample, ${\hat{y}}_{followup}$ is the risk score predicted by the response model, $E_{i}$ refers to the follow-up status (1 for death and 0 for survival) for patient *i*, and $T_{i}$ denotes the survival time for patient *i*. The total loss can be defined as,

$$L=L_{pcr}\left({\hat{y}}_{tab}\right)+L_{pcr}\left({\hat{y}}_{img}\right)+L_{pcr}\left({\hat{y}}_{tct}\right)+L_{pcr}\left({\hat{y}}_{PoE}\right)+L_{pcr}\left({\hat{y}}_{correct}\right)+L_{cox}\left({\hat{y}}_{tab}\right)+L_{cox}\left({\hat{y}}_{img}\right)+L_{cox}\left({\hat{y}}_{tct}\right)+L_{cox}\left({\hat{y}}_{PoE}\right)+L_{cox}\left({\hat{y}}_{correct}\right)+\lambda_{KL}\left(L_{KL}\left(z_{tab}\right)+L_{KL}\left(z_{img}\right)+L_{KL}\left(z_{txt}\right)+L_{KL}\left(z_{PoE}\right)\right)$$

After each training epoch, the optimal checkpoint for the model was determined by selecting the highest AUC of pCR prediction and the C-index of the overall survival in the validation set, respectively.

### Implementation details

The model was developed with PyTorch 2·1·2, and experiments were conducted on the NVIDIA Quadro RTX A6000 GPU. The model was trained using the AdamW optimizer with an initial learning rate of 0·0001. The training runs for 100 epochs for CLIP with a batch size of 16. After that, the weights of encoders were frozen, and the feature vectors corresponding to the modalities were calculated to train the response model, running for 500 steps with a batch size of 655. We divided the NKI cohort randomly into a training set and an internal validation set in a 1:1 ratio based on the patient level. The five best model checkpoints were selected based on five-fold cross-validation on the training set, and then the average pCR and risk scores of the five checkpoints were calculated on the NKI internal validation set and the DUKE external validation cohort for validation.

### Visualization and explainability

To elucidate the predictive patterns of pCR and risk scores, we visualized the focus of the model for different inputs. The permutation importance of input variables were calculated to determine their importance in clinical data. The heatmaps using the gradient-weighted class activation mapping (Grad-CAM) technique were generated to show the spatial attention of the model on DCE-MRI images. The correlation heatmap of the attention head in the text encoder was utilized to identify keywords and provide oral explanation from medical reports that are highly relevant to pCR and risk prediction. Additionally, Gephi was used to create visualizations of the co-occurrences of tokens with the top 100 attention weights in medical reports. The ForceAtlas2 algorithm was used to plot the network, with tokens represented as nodes and correlations as connecting edges between the tokens. The strength of the correlation is reflected in the weight of the edge. The nodes with only connecting edges with correlations lower than 1000 were omitted. To gain insights into how the model leveraged images and reports to correct inefficient regimens based on clinical data to achieve personalized recommendations, we generated density histograms of the true posterior distributions of prognosis for these correction samples.

### Statistics

Statistical analysis was done using Python 3·11·3 and R 4·2·2. The scikit-learn package was used to calculate the AUC, and a bootstrap method with 1000 replications was applied to generate 95% confidence intervals (CI). The dcurves package was used for decision curve analysis. The survcomp package was used to calculate the C-index and the corresponding 95% CI. The Kaplan–Meier method and the log-rank test were done with survival and survminer packages. All statistical tests were two-sided, and p < 0·05 indicated statistical significance.

### Role of the funding source

The funders of the study had no role in study design, data collection, data analysis, data interpretation, or writing of the report.

## Results

### Response and survival prediction

**Fig. 2:**
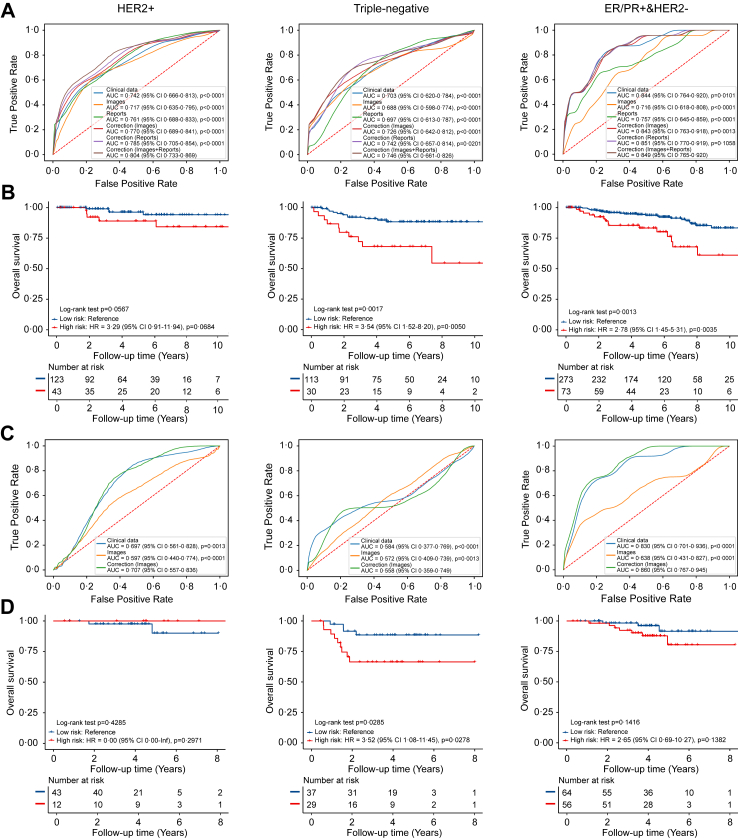
Prognosis performance of pathological complete response (pCR) scores and risk scores predicted by response models in the NKI internal validation set and DUKE external validation cohort. ROC curves for pCR status prediction are presented for HER2+, triple-negative, and ER/PR+&HER2− in NKI validation set (A) and DUKE cohort (C). For survival analysis, patients were stratified by predicted risk score as high risk or low risk. The 25% cutoff of the predicted risk score of the training set was used as the cutoff (−0·394, 0·424, 0·570). Kaplan–Meier curves for overall survival are presented for HER2+, triple-negative, and ER/PR+&HER2− in NKI validation set (B) and DUKE cohort (D). The p-values in the ROC analysis were derived using t-tests comparing each method against our proposed approach.

The developed multi-modal probabilistic response model for personalized NAT regimen recommendation (see Fig. 1) was trained on 655 patients using five-fold cross-validation and validated on 896 patients (655 for internal validation and 241 for external validation). Patient characteristics across cohort and NAT regimens are summarized in Table 1, with comparing distribution of clinical features between NKI and DUKE cohorts (Appendix Table S1), between lower-toxicity and higher-toxicity regimens (Appendix Table S2), and between training and validation sets (Appendix Table S3). pCR rates were 61·9% for HER2+, 53·5% for triple-negative, and 7·1% for ER/PR+&HER2− in the NKI cohort, 36·4%, 24·2%, and 6·5% in the DUKE cohort. Median overall follow-up times were 4·4 years (IQR 2·3) for HER2+, 4·6 (IQR 2·2) years for triple-negative, and 4·9 (IQR 2·6) years for ER/PR+&HER2− in the NKI cohort, 4·4 (IQR 3·2), 3·7 (IQR 1·8), and 4·1 (IQR 3·2) years in the DUKE cohort. As shown in Fig. 2 and Appendix Table S4, the model predicted the prognosis performance of patients after receiving the factual NAT regimen, achieving pCR prediction AUCs of 0·804 (95% CI 0·733–0·869), overall survival C-index of 0·695 (0·316–0·918) for HER2+, AUC of 0·746 (0·661–0·826) and C-index of 0·714 (0·474–0·874) for triple-negative, and AUC of 0·849 (0·765–0·920) and C-index of 0·612 (0·413–0·780) for ER/PR+&HER2− in the NKI internal validation set. Patients were divided into high-risk and low-risk groups by the 25% cutoffs of the negative predicted risk score (−0·394 for HER2+, 0·424 for triple-negative, and 0·570 for ER/PR+&HER2−) within the NKI training set. Patients in the high-risk group had worse outcomes than patients in the low-risk group in the NKI internal validation set. Similar validation in the DUKE cohort yielded pCR AUCs of 0·707 (0·557–0·836), 0·558 (0·359–0·749), and 0·860 (0·767–0·945), and C-indexes of 0·731 (0·133–0·980), 0·753 (0·460–0·916), and 0·638 (0·299–0·879) for each molecular subtype group, respectively. In summary, these results suggest that the multi-modal model can help predict the outcome of patients with breast cancer treated with their factual NAT regimens.

**Table 1:** Patient characteristics with different neoadjuvant therapy (NAT) regimens in multiple data cohorts.

Cohorts	NKI (n = 1310)					DUKE (n = 241)		
	R1	R2	R3	R4	R5	R2	R4	R5
Demographics								
Number of patients	443	394	186	147	140	168	55	18
Age (±SD)	48·3 ± 10·8	49·2 ± 11·5	48·6 ± 11·8	49·2 ± 11·5	60·6 ± 11·6	49·0 ± 11·2	47·5 ± 11·0	63·3 ± 7·9
Weight (±SD)	72·6 ± 10·5	71·9 ± 11·8	72·0 ± 10·0	71·7 ± 11·1	72·3 ± 11·6	–	–	–
Menopause status								
Premenopausal	145 (33%)	172 (44%)	56 (30%)	56 (38%)	26 (19%)	98 (58%)	32 (58%)	2 (11%)
Perimenopausal	17 (4%)	26 (7%)	10 (5%)	15 (10%)	8 (6%)	0 (0%)	0 (0%)	0 (0%)
Postmenopausal	100 (23%)	141 (36%)	40 (22%)	46 (31%)	74 (53%)	69 (41%)	23 (42%)	16 (89%)
Missing	181 (41%)	55 (14%)	80 (43%)	30 (20%)	32 (23%)	1 (1%)	0 (0%)	0 (0%)
Stage								
1	20 (5%)	50 (13%)	40 (22%)	3 (2%)	38 (27%)	13 (8%)	3 (5%)	3 (17%)
2	322 (73%)	263 (67%)	98 (53%)	106 (72%)	100 (71%)	101 (60%)	39 (71%)	13 (72%)
3	101 (23%)	81 (21%)	48 (26%)	38 (26%)	2 (1%)	54 (32%)	13 (24%)	2 (11%)
Pathological T stage								
T1	86 (19%)	100 (25%)	64 (34%)	24 (16%)	54 (39%)	39 (23%)	9 (16%)	5 (28%)
T2	262 (59%)	220 (56%)	94 (51%)	94 (64%)	76 (54%)	90 (54%)	34 (62%)	11 (61%)
T3	83 (19%)	67 (17%)	23 (12%)	24 (16%)	10 (7%)	31 (18%)	10 (18%)	2 (11%)
T4	12 (3%)	7 (2%)	5 (3%)	5 (3%)	0 (0%)	8 (5%)	2 (4%)	0 (0%)
Pathological N stage								
N0	160 (36%)	180 (46%)	72 (39%)	60 (41%)	106 (76%)	64 (38%)	23 (42%)	14 (78%)
N1	235 (53%)	167 (42%)	84 (45%)	66 (45%)	34 (24%)	68 (40%)	27 (49%)	2 (11%)
N2	5 (1%)	2 (1%)	2 (1%)	1 (1%)	0 (0%)	23 (14%)	4 (7%)	1 (6%)
N3	43 (10%)	45 (11%)	28 (15%)	20 (14%)	0 (0%)	13 (8%)	1 (2%)	1 (6%)
Hormone receptor								
ER/PR+	324 (73%)	229 (58%)	115 (62%)	97 (66%)	140 (100%)	102 (61%)	37 (67%)	18 (100%)
ER/PR−	119 (27%)	165 (42%)	71 (38%)	50 (34%)	0 (0%)	66 (39%)	18 (33%)	0 (0%)
HER2+	0 (0%)	0 (0%)	186 (100%)	147 (100%)	0 (0%)	0 (0%)	55 (100%)	0 (0%)
HER2−	443 (100%)	394 (100%)	0 (0%)	0 (0%)	140 (100%)	168 (%)	0 (0%)	18 (100%)

R1: Anthracycline backbone. R2: Anthracycline plus taxane backbone. R3: Single HER2 antibody. R4: Multiple HER2 antibodies. R5: Hormone therapy. Data are n (%). SD: standard deviation.

ROC curve analysis and Kaplan–Meier analysis in Fig. 2 further demonstrated that incorporating image- and report-based information can correct and improve predictions over clinical data alone. For the NKI internal validation set, the AUCs for multi-modal-based pCR prediction were significantly higher than those based on clinical data, for HER2+ of 0·742 (0·666–0·813; p < 0·0001), triple-negative of 0·703 (0·620–0·784; p < 0·0001), and ER/PR+&HER2− of 0·844 (0·764–0·920; p = 0·0101). The hazard ratios (HR) of the predicted score-based risk group for overall survival were 3·29 (0·91–11·94; p = 0·0684) for HER2+, 3·54 (1·52–8·20; p = 0·0050) for triple-negative, and 2·78 (1·45–5·31; p = 0·0035) for ER/PR+&HER2−, exceeding results of Cox proportional hazards with regularization (Appendix Figure S19). A statistically significant difference was observed for the triple-negative and ER/PR+&HER2− subtypes, while the result for HER2+ did not reach statistical significance. The external validation cohort demonstrated similar improvements, with AUCs of 0·697 (0·561–0·828; p = 0·0013), 0·584 (0·377–0·769; p < 0·0001), and 0·830 (0·701–936; p < 0·0001) for HER2+, triple-negative, and ER/PR+&HER2−, indicating the robustness of the multi-modal model. Additional analyses based on different clinical subgroups are presented in Appendix Figures S6–S11, demonstrating the high performance on risk stratification of our model. Our deep learning-based risk stratification providing more accurate prognostic assessments than molecular subtypes alone (see Appendix Figure S12).

### Model interpretability

**Fig. 3:**
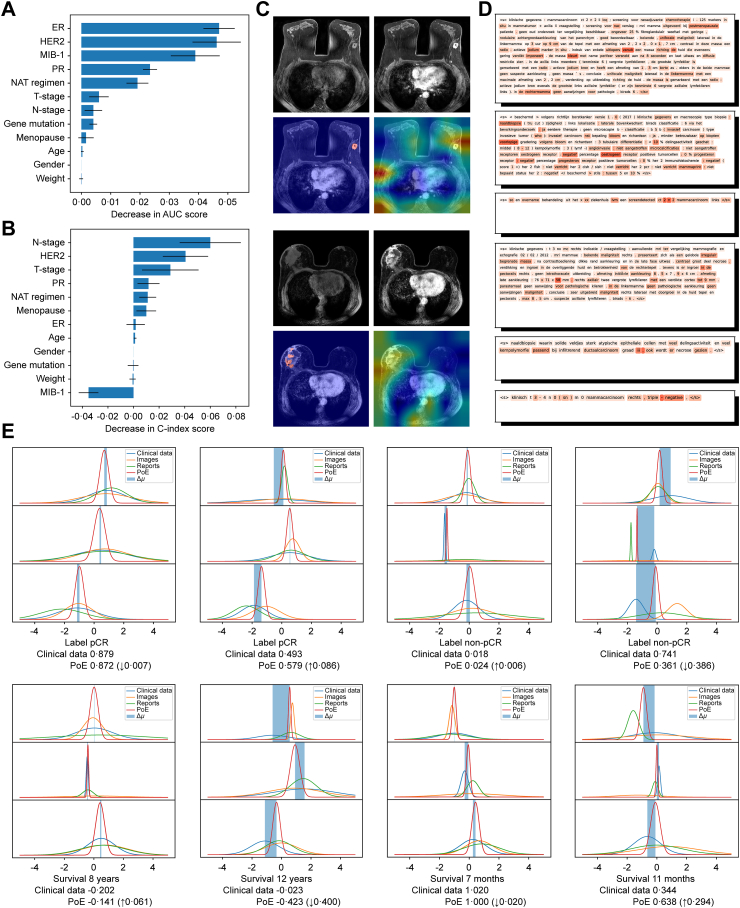
Visualization of model interpretability in the NKI internal validation set. The permutation importances for each clinical variable in predicting pathological complete response (pCR) (A) and survival (B) are listed. Data were shown as mean ± standard deviation. The gradient-weighted class activation mapping (Grad-CAM) heatmaps of the dynamic contrast enhanced (DCE)-MRI are displayed in (C) in the following order: pre-contrast DCE-MRI at the top left, post-contrast DCE-MRI at the top right, segmentation mask at the bottom left, and heatmap at the bottom right. The correlation heatmaps of the reports are displayed in (D) in the following order from top to bottom: radiology report, pathology report, and medical record for each patient. The density histograms of the prognosis distributions are presented in (E). Δμ indicates the correction of the mean between Product-of-Experts (PoE) and clinical data.

The interpretability of the model was examined through visualization in Fig. 3. Among clinical data variables, the receptor status (ER, PR, and HER2) achieved highest permutation importance for pCR prediction, while the N-stage played the most important role for survival prediction. For image-based information, the heatmap indicated that the model effectively focused on the tumor regions, attributed to the attention provided by the input tumor segmentation mask. Report-based information emphasized text details such as tumor description in radiology reports (e.g., “indruk van enkele uitlopers vanuit een massa richting de huid die eveneens gering verdikt imponeert”/“impression of a few extensions from a mass towards the skin that also appears slightly thickened”), tumor receptor status in pathology reports (e.g., “percentage oestrogeen receptor positieve tumorcellen”/“percentage of estrogen receptor positive tumor cells”), and the tumor stage in medical records (e.g., “ct2n2”). Density histograms demonstrated how image- and report-based information corrected clinical data-based predictions. Greater deviations in the mean of the PoE distribution from the mean of the clinical data distribution are related to more significant prediction corrections. More visualization for image and report encoders can be found in Appendix Figures S13 and S14, and the visualization of word co-occurrence for tokens with high attention weights is shown in Appendix Table S5 and Figure S15. Feature impact based on SHAP analysis is shown in Appendix Figure S20 and t-SNE of data distribution is shown in Appendix Figure S18.

### Personalized regimen recommendation

**Fig. 4:**
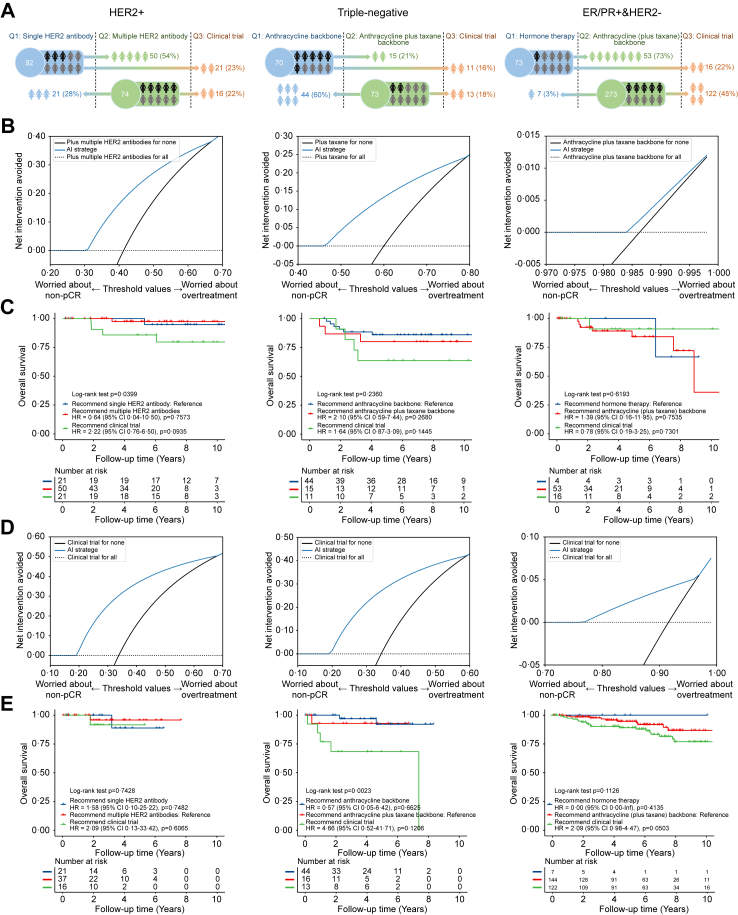
Benefits of artificial intelligence (AI) recommendations in different factual regimen subgroups of patients in the NKI internal validation set. Our AI recommendation system can recommend lower/higher-toxicity regimens or clinical trials for different patient populations (A). Decision curve for AI recommendation on patients with factual regimens of lower-toxicity regimens (B) and patients with factual regimens of higher-toxicity regimens (D). Kaplan–Meier survival curve for patients applying model-based three-category regimen recommendation on patients with factual regimens of lower-toxicity regimens (C) and patients with factual regimens of higher-toxicity regimens (E). The p-values in the Kaplan–Meier survival curve analysis were derived using log-rank tests comparing each regimen against the reference regimen.

We further assessed the patient benefits of applying our three-category regimen recommendations (see Appendix Table S6) in the NKI internal validation set in Fig. 4. Regarding achieving short-term outcome pCR, for HER2+ patients treated with single HER2 antibody regimens, applying regimen recommendations by multi-modal model prevented 20% of patients from overtreatment compared with adding multiple HER2 antibodies to everyone, when non-pCR was 1·5 times more severe than overtreatment. For HER2+ patients treated with multiple HER2 antibodies regimens or triple-negative patients treated with anthracycline plus taxane backbone, applying regimen recommendations prevented 40% and 35% of patients from overtreatment compared with enrolling clinical trials with active drugs to everyone. In terms of long-term outcome overall survival, while the model identified patient subgroups where alternative treatment strategies, including clinical trial enrollment, might be warranted, the HRs for these groups had wide confidence intervals crossing 1, indicating no statistically significant difference in prognosis. For HER2+ patients treated with single HER2 antibody regimens, the study did not find a statistically significant difference between the group for whom enrolling clinical trials was recommended by the model and the group did not, as reflected by an HR of 2·22 (0·76–6·50). For triple-negative patients treated with anthracycline backbone regimens, the group for whom the model recommended supplemental taxane had no statistically significant difference in prognosis for cases where taxane was not recommended by the model, as reflected by an HR of 2·10 (0·59–7·44) for death. For ER/PR+&HER2− patients treated with hormone therapy regimens, no statistically significant difference was observed between the group for whom chemotherapy was recommended by the model and the group did not, as reflected by an HR of 1·39 (0·16–11·95) for death. Likewise, for HER2+ patients treated with multiple HER2 antibodies regimens, triple-negative patients treated with anthracycline plus taxane backbone, or ER/PR+&HER2− patients treated with anthracycline (plus taxane) backbone, the risk group for whom a clinical trial was recommended by the model, because current therapy was judged to be likely insufficient, had no statistically significant difference in prognosis for other recommended regimens, as reflected by HRs of 2·09 (0·13–33·42), 4·66 (0·52–41·71), and 2·09 (0·98–4·47). For the external validation cohort, the NAT regimen recommendation based on the multi-modal model still brought stable benefits to patients in Appendix Figure S16, reflected by HRs of 0·17 (0·03–0·083) and 2·01 (0·61–6·61) for triple-negative patients.

## Discussion

In this study, we developed a multi-modal-based NAT outcome prediction system to evaluate the outcomes of different potential NAT regimens to work towards personalized regimen recommendations. Our response model achieved promising predictive performance in a cohort from the Netherlands and was consistently validated in another external validation cohort. The three-category regimen recommendation based on model-predicted pCR and risk score can effectively stratify patients by their prognosis.

Our model has been validated on multiple cohorts and shows consistent performance. Image- and report-based information showed significant correction to predictions relying solely on clinical data, demonstrating the potential of leveraging multi-modal information for personalized predictions. We observed that in NKI and DUKE cohorts, the performance of pCR prediction using clinical data for patients with different molecular subtypes was different, with ER/PR+&HER2− having the best prediction performance, followed by HER2+, and triple-negative being the worst. After correction by image and report information, the prediction performance of HER2+ and triple-negative in the NKI cohort was significantly improved, while ER/PR+&HER2− improvement was limited. This shows that some information helpful for pCR prediction that is lacking in clinical data can be extracted from images and reports. In the DUKE cohort, the prediction results of HER2+ and ER/PR+&HER2− also improved after correction, but the performance in triple-negative breast cancer was poor, potentially because these tumors have strong heterogeneity and are challenging to evaluate.^28^ The survival prediction had different accuracy for each molecular subtype. In both the NKI and DUKE cohorts, triple-negative patients had the highest C-index, followed by HER2+ patients. The worst performance was seen in patients with ER/PR+&HER2−. Kaplan–Meier analysis showed that predicted risk scores were most effective at stratifying triple-negative patients, followed by ER/PR+&HER2−, with HER2+ patients exhibiting minimal distinction due to improved survival from HER2 targeted therapies.^29^^,^^30^

Despite its effectiveness, challenges remain in ensuring model interpretability for clinical applications. We visually analyzed the areas of interest of the model for each modal and demonstrated the process of integrating multi-modal information. Our findings indicate that the model places greater emphasis on the molecular subtypes and staging information of the tumor in clinical data, tumor-surrounding tissue in images, and detailed tumor descriptions in medical reports for breast cancer patients, which is consistent with previous research.^14^ We also discovered that integrating multi-modal data can reduce variance in the posterior distributions of specific dimensions, improving prediction accuracy. Furthermore, medical reports are proposed to serve as a valuable multi-modal data source that enhances model performance by providing supplementary pathological and historical data and leveraging expert knowledge via CLIP alignment to improve image feature extraction.

Guideline-based NAT regimen selection may not be entirely appropriate for individuals and may lead to over- or under-treatment. Our model enables individualized treatment by predicting outcomes for each NAT regimen and comparing their efficacy. For patients applying a particular regimen, the three-category recommendation system shows the potential of our model to recommend clinical trials for patients who do not achieve pCR using the factual regimen. Patients with predicted better outcomes may be advised to try lower-toxicity regimens, and others could be recommended to use higher-toxicity regimens. This personalized approach may reduce inefficiency or overtreatment caused by regimens generally determined according to cancer stage and molecular subtypes without fully considering the individual variants of patients. Furthermore, the decision-making process is influenced by the selected risk score threshold. Adapting the threshold based on molecular subtypes can mitigate potential model bias, ensuring more accurate and equitable risk stratification across different patient groups.

Our study has several limitations. We acknowledge that the data were collected over a long time span, which, while not equivalent to randomized clinical trials, provides valuable observational diversity that enables modeling and comparison of different therapies in real-world settings. However, changes in diagnostic and therapeutic tools other than those modeled in this study are not accounted for and may have influenced the results. For instance, the distinction between HER2+&ER− and HER2+&ER+ subtypes, now increasingly recognized to have different biological behavior and treatment responses, was not separately addressed in the therapy group. Additionally, our analysis did not incorporate more recent molecular and omic-level classifications, which are likely to refine breast cancer subtypes and treatment strategies further as the understanding of biology and immunologic therapies evolves. The observed pCR rate in the external validation set is lower than in the in-house dataset. This might be due to temporal changes in therapy, but it may also indicate that the subgroups are not completely comparable. The fact, however, that the algorithm still performs well in the entire external validation set shows that the model is relatively robust towards such variations in the data. Despite external validation, the datasets were limited in size and retrospectively collected, with the external cohort having fewer NAT regimens (there was only one regimen for HER2+ and triple-negative), potentially affecting performance evaluation. Variability in clinical practices and changes in NAT regimens over time were also not fully accounted for. Some patients may have received regimens dictated by clinical trial protocols rather than optimal treatment for the general population. In triple-negative cancers, considering anthracycline backbone as the lower-toxicity regimen might not be entirely appropriate as it may be given longer, and a lighter regimen might nowadays consist of taxane–cyclophosphamide (TC). However, only historical real-life data was used for modeling dating from the time that AC was a common regimen. Another limitation is that our approach relied solely on pre-treatment MRI, without accounting for early treatment response, which is increasingly used in clinical decision-making to personalize therapy. The median follow-up time was relatively short, particularly for patients with ER/PR+&HER2− breast cancer. Consequently, our survival analyses may underestimate long-term relapse risk in this subgroup. Additionally, the impact of adjuvant therapy on overall survival was not considered. While our model can recommend lower-toxicity regimens (e.g., single HER2 antibody) for certain patients treated with higher-toxicity regimens (e.g., multiple HER2 antibodies), clinical validation is necessary to ensure comparable outcomes. There is no guarantee that these patients will achieve the same outcome using the recommended regimen, which may lead to an overestimation of model power. Therefore, more extensive prospective trials assessing the true sensitivity and specificity of the recommendations are needed to validate our results.

In conclusion, our multi-modal response model integrates radiological, pathological, and clinical information to predict pCR status and estimate long-term survival outcomes under different NAT regimens. While the model demonstrated improved predictive performance over clinical variables alone, including molecular subtypes, its ability to stratify patient prognosis did not show statistically significant associations in survival outcomes. These findings may serve as a preliminary step toward aiding clinical trial cohort selection, but further validation in prospective, controlled studies is essential, particularly to determine whether model-informed low-toxicity NAT strategies can maintain efficacy while reducing overtreatment.

## Contributors

LH and TT conceived the study. LH wrote the manuscript. LH and TZ processed the data, performed the experiment, and performed the analysis. AD, AV, KP, MK, GS, and RM contributed clinical expertise. TT and RM supervised the work. LH, TZ, AD, AV, KP, MK, GS, YG, XW, CL, XL, JT, TT, and RM contributed to interpreting the results and editing the final manuscript. LH, TZ, AD, AV, KP, MK, GS, YG, XW, CL, XL, JT, TT, and RM had full access to the data. LH, TZ, and RM directly accessed and verified the underlying data in the study. All authors accept the final responsibility to submit for publication and take responsibility for the contents of the manuscript.

## Data sharing statement

The NKI dataset is private and not publicly available to protect the privacy of the patients. The DUKE dataset is publicly available at https://doi.org/10.7937/TCIA.e3sv-re93. Excel file containing source data included in the main figures and tables can be found in the Source Data File in the article. All codes for this study are open source and available at https://github.com/fiy2W/MORM. It should only be used for non-commercial and academic research. Additional information required to reanalyze the data reported in this work was provided by the corresponding study Principal Investigators. Access can be obtained by request to the corresponding author, keeping legal prerequisites in mind.

## Declaration of interests

The authors declare no competing interests.
