# Supplementary material for: Exploring personalized neoadjuvant therapy selection strategies in breast cancer: an explainable multi-modal response model
Source: eClinicalMedicine. 2025 Jul 17;86:103356. doi: 10.1016/j.eclinm.2025.103356 (PMC12303063; doi:10.1016/j.eclinm.2025.103356)
Supplement: Appendix Figures and Tables [file mmc1.pdf]

A Supplementary tables

Table S1. Comparison of clinical feature distributions across different datasets with the same NAT regimens. The two-sided independent samples T-test is applied for age, and the Chi-square test is utilized for categorical variables. R2: Anthracycline plus taxane backbone. R4: Multiple HER2 antibodies. R5: Hormone therapy. Menopause status: Premenopausal/Postmenopausal. Stage: 1/2/3. Pathological T stage: T1/T2/T3/T4. Pathological N stage: N0/N1/N2/N3. Hormone receptor: HR+/HR-/HER2+/HER2-.

Group	Age	Menopause status	Stage	Pathological T stage	Pathological N stage	Hormone receptor
NKI-R2	49.2 ± 11.5	172/141	50/263/81	100/220/67/7	180/167/2/45	229/165/0/394
DUKE-R2	49.0 ± 11.2	98/69	13/101/54	39/90/31/8	64/68/23/13	102/66/0/168
p-value	0.8492	0.4913	0.0069	0.2207	<0.0001	0.6325
NKI-R4	49.2 ± 11.5	56/46	3/106/38	24/94/24/5	60/66/1/20	97/50/147/0
DUKE-R4	47.5 ± 11.0	32/23	3/39/13	9/34/10/2	23/27/4/1	37/18/55/0
p-value	0.3452	0.8208	0.4369	0.9897	0.0057	0.9960
NKI-R5	60.6 ± 11.6	26/74	38/100/2	54/76/10/0	106/34/0/0	140/0/0/140
DUKE-R5	63.3 ± 7.9	2/16	3/13/2	5/11/2/0	14/2/1/1	18/0/0/18
p-value	0.3396	0.2864	0.0372	0.6178	0.0008	1.0000

Table S2. Comparison of clinical feature distributions between patients treated with lower-toxicity and higher-toxicity regimens. The two-sided independent samples T-test is applied for age and weight, and the Chi-square test is utilized for categorical variables. R1: Anthracycline backbone. R2: Anthracycline plus taxane backbone. R3: Single HER2 antibody. R4: Multiple HER2 antibodies. R5: Hormone therapy. Menopause status: Premenopausal/Postmenopausal. Stage: 1/2/3. Pathological T stage: T1/T2/T3/T4. Pathological N stage: N0/N1/N2/N3. Hormone receptor: HR+/HR-/HER2+/HER2-.

Group	Age	Weight	Menopause status	Stage	Pathological T stage	Pathological N stage	Hormone receptor
NKI-R1	48.3±10.8	72.6±10.5	145/100	20/322/101	86/263/83/12	160/235/5/43	324/119/0/443
NKI-R2	49.2±11.5	71.9±11.8	172/141	50/263/81	100/220/67/7	180/167/2/45	229/165/0/394
p-value	0.2435	0.3641	0.3600	<0.0001	0.1763	0.0108	<0.0001
NKI-R3	48.6±11.8	72.0±10.0	56/40	40/98/48	64/94/23/5	72/84/2/28	115/71/186/0
NKI-R4	49.2±11.5	71.7±11.1	56/46	3/106/38	24/94/24/5	60/66/1/20	97/50/147/0
p-value	0.6416	0.7959	0.7313	<0.0001	0.0032	0.9494	0.5037
NKI-R2	49.2±11.5	71.9±11.8	172/141	50/263/81	100/220/67/7	180/167/2/45	229/165/0/394
NKI-R5	60.6±11.6	72.3±11.6	26/74	38/100/2	54/76/10/0	106/34/0/0	140/0/0/140
p-value	<0.0001	0.7294	<0.0001	<0.0001	0.0013	<0.0001	<0.0001
DUKE-R2	49.0±11.2	-	98/69	13/101/54	39/90/31/8	64/68/23/13	102/66/0/168
DUKE-R5	63.3±7.9	-	2/16	3/13/2	5/11/2/0	14/2/1/1	18/0/0/18
p-value	<0.0001	-	0.0003	0.1174	0.6449	0.0131	0.0023

Table S3. Patient characteristics with different NAT regimens between the training and internal validation set in the NKI cohort. The two-sided independent samples T-test is applied for age and weight, and the Chi-square test is utilized for categorical variables. R1: Anthracycline backbone. R2: Anthracycline plus taxane backbone. R3: Single HER2 antibody. R4: Multiple HER2 antibodies. R5: Hormone therapy. Data are n (%). SD: standard deviation. T: training set. V: internal validation set.

Cohorts	R1			R2			R3			R4			R5		
	T	V		T	V		T	V		T	V		T	V	
Demographics															
Number of patients	205	238		216	178		94	92		73	74		67	73	
Age (±SD)	48.9±11.0	47.9±10.7	($p = 0.3335$)	49.9±11.7	48.4±11.2	($p = 0.1974$)	48.5±11.8	48.7±11.8	($p = 0.9081$)	48.5±11.4	50.0±11.6	($p = 0.4305$)	61.7±10.8	59.5±12.3	($p = 0.2645$)
Weight (±SD)	72.0±10.7	73.1±10.3	($p = 0.2716$)	71.8±12.2	72.2±11.2	($p = 0.7370$)	72.2±9.0	71.8±10.9	($p = 0.7850$)	72.1±10.6	71.3±11.6	($p = 0.6632$)	72.7±11.1	71.8±12.1	($p = 0.6482$)
Menopause status			($p = 0.4000$)			($p = 0.8009$)			($p = 0.4817$)			($p = 0.4492$)			($p = 0.1770$)
Premenopausal	70	75		94	78		33	23		29	27		9	17	
Perimenopausal	10	7		12	14		4	6		10	5		3	5	
Postmenopausal	49	51		80	61		19	21		20	26		35	39	
Missing	76	105		30	25		38	42		14	16		20	12	
Stage			($p = 0.6015$)			($p = 0.5478$)			($p = 0.3199$)			($p = 0.1799$)			($p = 0.1658$)
1	11	9		31	19		16	24		3	0		15	23	
2	145	177		141	122		52	46		53	52		52	48	
3	49	52		44	37		26	22		17	21		0	2	
Pathological T stage			($p = 0.9347$)			($p = 0.5466$)			($p = 0.3203$)			($p = 0.7538$)			($p = 0.0679$)
T1	42	44		61	39		28	36		14	10		23	31	
T2	121	141		116	104		51	43		45	49		42	34	
T3	37	46		35	32		11	12		11	13		2	8	
T4	5	7		4	3		4	1		3	2		0	0	
Pathological N stage			($p = 0.9386$)			($p = 0.2586$)			($p = 0.3974$)			($p = 0.6033$)			($p = 0.2027$)
N0	73	87		93	87		34	38		30	30		47	59	
N1	109	126		97	70		44	40		34	32		20	14	
N2	3	2		0	2		0	2		1	0		0	0	
N3	20	23		26	19		16	12		8	12		0	0	
Hormone receptor			($p = 0.2313$)			($p = 0.8305$)			($p = 0.3073$)			($p = 0.9085$)			($p = 1.0000$)
ER/PR+	156	168		124	105		62	53		49	48		67	73	
ER/PR-	49	70		92	73		32	39		24	26		0	0	
HER2+	0	0		0	0		94	92		73	74		0	0	
HER2-	205	238		216	178		0	0		0	0		67	73	

Table S4. **C-index of overall survival based on the predicted risk scores for the ablation study in the NKI-internal validation set and DUKE external validation cohort.**

	Method	NKI			DUKE		
		HER2+	Triple negative	ER/PR+&HER2-	HER2+	Triple negative	ER/PR+&HER2-
(A)	NFS + FS	**0.619 (0.261-0.882)**	0.498 (0.282-0.715)	0.463 (0.281-0.656)	0.358 (0.035-0.896)	0.481 (0.232-0.739)	0.416 (0.151-0.740)
	Wash-in	0.499 (0.184-0.815)	0.570 (0.342-0.772)	0.393 (0.224-0.592)	**0.612 (0.095-0.959)**	0.593 (0.319-0.820)	0.493 (0.198-0.793)
	Normalized FS	0.599 (0.248-0.872)	**0.664 (0.426-0.840)**	**0.549 (0.355-0.729)**	0.433 (0.050-0.917)	**0.692 (0.403-0.882)**	**0.606 (0.275-0.862)**
(B)	DCE	0.517 (0.194-0.826)	0.465 (0.255-0.688)	0.537 (0.344-0.719)	0.537 (0.075-0.944)	0.613 (0.335-0.833)	0.552 (0.237-0.830)
	DCE + mask (concat)	0.527 (0.201-0.832)	0.545 (0.320-0.753)	**0.618 (0.418-0.784)**	**0.582 (0.087-0.953)**	0.540 (0.277-0.783)	**0.626 (0.290-0.873)**
	DCE + mask (attention)	**0.599 (0.248-0.872)**	**0.664 (0.426-0.840)**	0.549 (0.355-0.729)	0.433 (0.050-0.917)	**0.692 (0.403-0.882)**	0.606 (0.275-0.862)
(C)	Radiology report	0.574 (0.231-0.858)	0.640 (0.404-0.824)	0.551 (0.357-0.731)	-	-	-
	Pathology report	**0.581 (0.235-0.863)**	0.480 (0.267-0.701)	0.554 (0.359-0.734)	-	-	-
	Medical record	0.563 (0.223-0.852)	0.369 (0.182-0.607)	0.542 (0.349-0.723)	-	-	-
	All medical reports	0.577 (0.233-0.860)	**0.658 (0.421-0.836)**	**0.565 (0.369-0.742)**	-	-	-
(D)	Initial weights	0.528 (0.202-0.832)	0.653 (0.416-0.833)	0.602 (0.403-0.772)	0.672 (0.113-0.970)	0.642 (0.359-0.851)	0.557 (0.241-0.833)
	CLIP	**0.695 (0.316-0.918)**	**0.714 (0.474-0.0.874)**	**0.612 (0.413-0.780)**	**0.731 (0.133-0.980)**	**0.753 (0.460-0.916)**	**0.638 (0.299-0.879)**
(E)	Clinical data	0.505 (0.187-0.819)	0.693 (0.453-0.860)	0.556 (0.362-0.735)	0.522 (0.071-0.940)	0.707 (0.416-0.891)	0.502 (0.204-0.799)
	Images	0.599 (0.248-0.872)	0.664 (0.426-0.840)	0.549 (0.355-0.729)	0.433 (0.050-0.917)	0.692 (0.403-0.882)	0.606 (0.275-0.862)
	Reports	0.577 (0.233-0.860)	0.658 (0.421-0.836)	0.565 (0.369-0.742)	-	-	-
	Correction (Images)	0.617 (0.260-0.881)	0.698 (0.458-0.863)	0.566 (0.370-0.743)	**0.731 (0.133-0.980)**	**0.753 (0.460-0.916)**	**0.638 (0.299-0.879)**
	Correction (Reports)	0.603 (0.250-0.873)	**0.715 (0.475-0.875)**	0.612 (0.412-0.779)	-	-	-
	Correction (Images+Reports)	**0.695 (0.316-0.918)**	0.714 (0.474-0.0.874)	**0.612 (0.413-0.780)**	-	-	-

Table S5. Keywords related to high co-occurrence words.

High co-occurrence words	Co-occurrence keywords	Weight
massa / mass	gespiculeerde / spiculated	0.111
	irregulaire / irregular	0.097
	dense / dense	0.083
	afgrensbaar / delimitable	0.079
	gelobuleerde / lobulated	0.067
	unifocale / unifocal	0.064
	heterogene / heterogeneous	0.063
	tweede / secondary	0.057
	nodulaire / nodular	0.053
	sprieterige / spiky	0.052
calcificaties / calcifications	echorijke / echoic	0.078
	dense / dense	0.064
	pleiomorfe / pleomorphic	0.060
	grove / coarse	0.060
	beiderzijds / bilateral	0.059
	geen / none	0.052
	kleine / small	0.051
	zichtbaar / visible	0.043
	heterogene / heterogeneous	0.042
	centrale / central	0.034
lymfklier / lymph node	een / one	0.058
	grote / large	0.051
	grootste / largest	0.038
	rechter / right	0.038
	gezien / seen	0.037
	kleine / small	0.036
	zichtbaar / visible	0.036
	prominente / prominent	0.029
	normale / normal	0.026
	axillair / axillary	0.026

Table S6. Risk stratification table for regimen recommendation of breast cancer.

Molecular subtype	Risk stratification	Risk score (O)	Recommended NAT regimen
HER2+	Low risk	O(Q1)≥-0·394; O(Q1)≥O(Q2)	Q1: Single HER2 antibody
	Middle risk	O(Q2)≥-0·394; O(Q1)<O(Q2)	Q2: Multiple HER2 antibodies
	High risk	O(Q1)<-0·394; O(Q2)<-0·394	Q3: Clinical trial
Triple-negative	Low risk	O(Q1)≥0·424; O(Q1)≥O(Q2)	Q1: Anthracycline backbone
	Middle risk	O(Q2)≥0·424; O(Q1)<O(Q2)	Q2: Anthracycline plus Taxane backbone
	High risk	O(Q1)<0·424; O(Q2)<0·424	Q3: Clinical trial
ER/PR+&HER2-	Low risk	O(Q1)≥0·570; O(Q1)≥O(Q2)	Q1: Hormone therapy
	Middle risk	O(Q2)≥0·570; O(Q1)<O(Q2)	Q2: Anthracycline (plus Taxane) backbone
	High risk	O(Q1)<0·570; O(Q2)<0·570	Q3: Clinical trial

B Supplementary figures

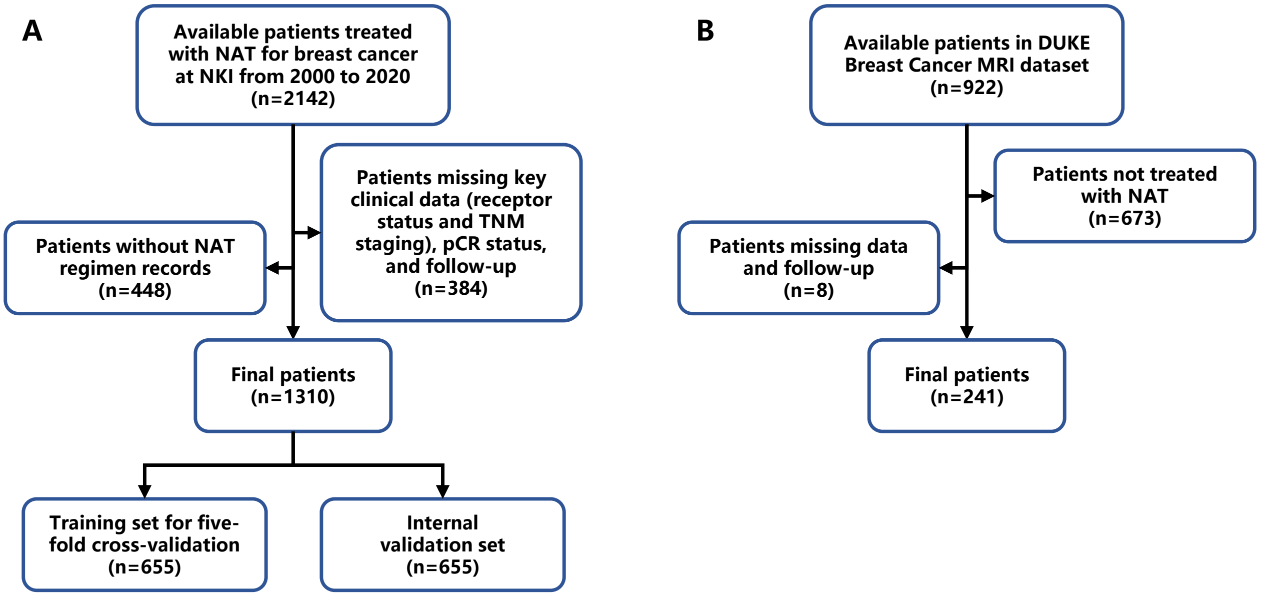

Figure S1. Flowchart of patient inclusion. (A) NKI cohort. (B) DUKE cohort.

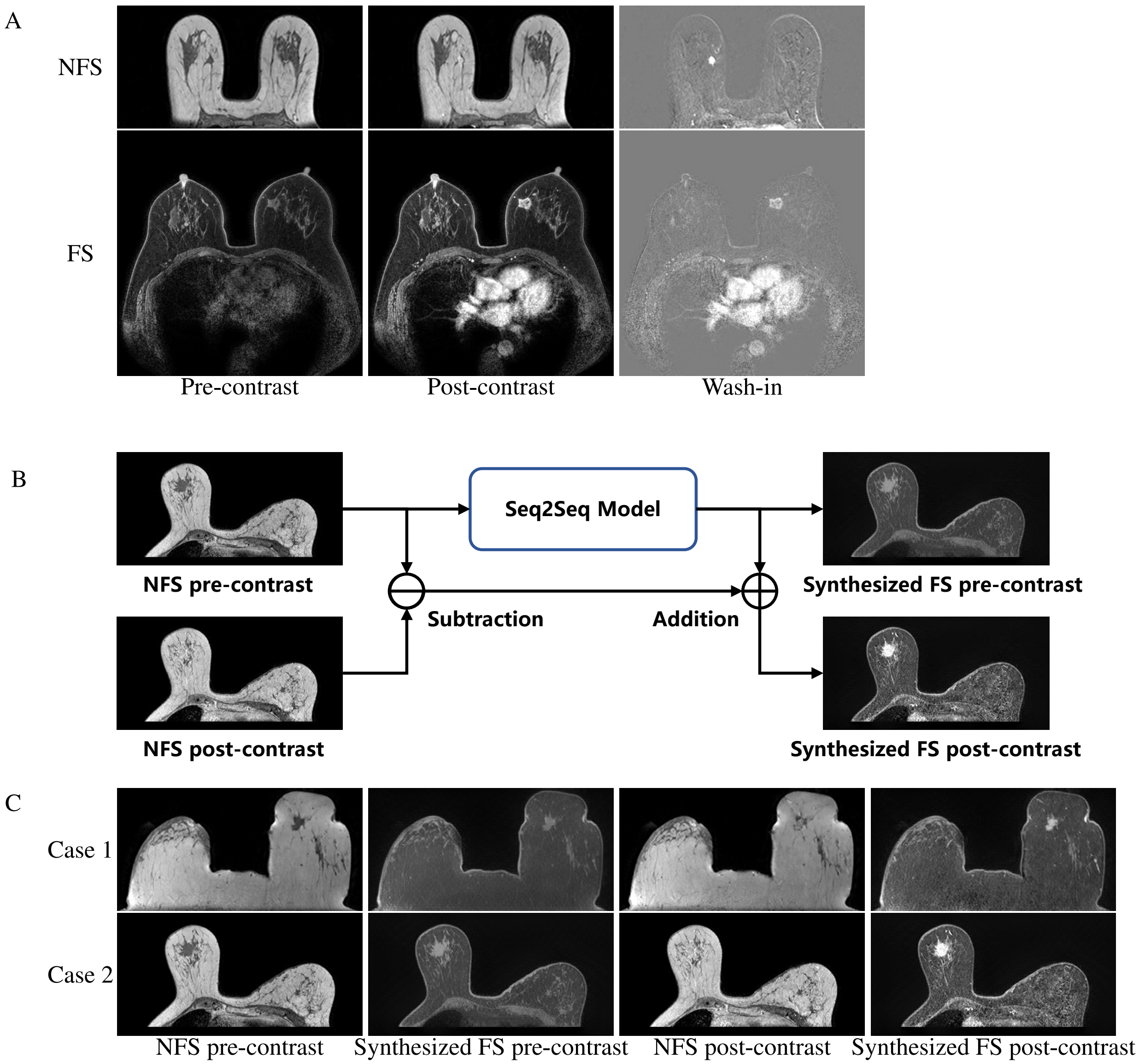

Figure S2. Examples of breast DCE-MRI and appearance normalization from the NKI cohort. (A) Example of non-fat-saturated (NFS) and fat-saturated (FS) DCE-MRI for breast cancer. The first row shows pre-contrast, post-contrast, and wash-in images of coronal scanned NFS DCE-MRI, while the second row shows images of transverse scanned FS DCE-MRI. The fat signal is suppressed in FS DCE-MRI, and the breast tissue appears dark, whereas breast tissue is white in NFS DCE-MRI. The thoracic cavity can be seen on a transverse scan, but it cannot be seen on a coronal scan. (B) Flowchart of synthesizing FS DCE-MRI from NFS images. The Seq2Seq model directly synthesizes the FS pre-contrast DCE-MRI from the NFS pre-contrast image. To maintain the enhancement of the NFS DCE-MRI, the subtraction between NSF post- and pre-contrast DCE-MRI is added to the synthesized FS pre-contrast image to get the synthesized FS post-contrast image. (C) Example of NFS DCE-MRI and corresponding synthesized FS DCE-MRI.

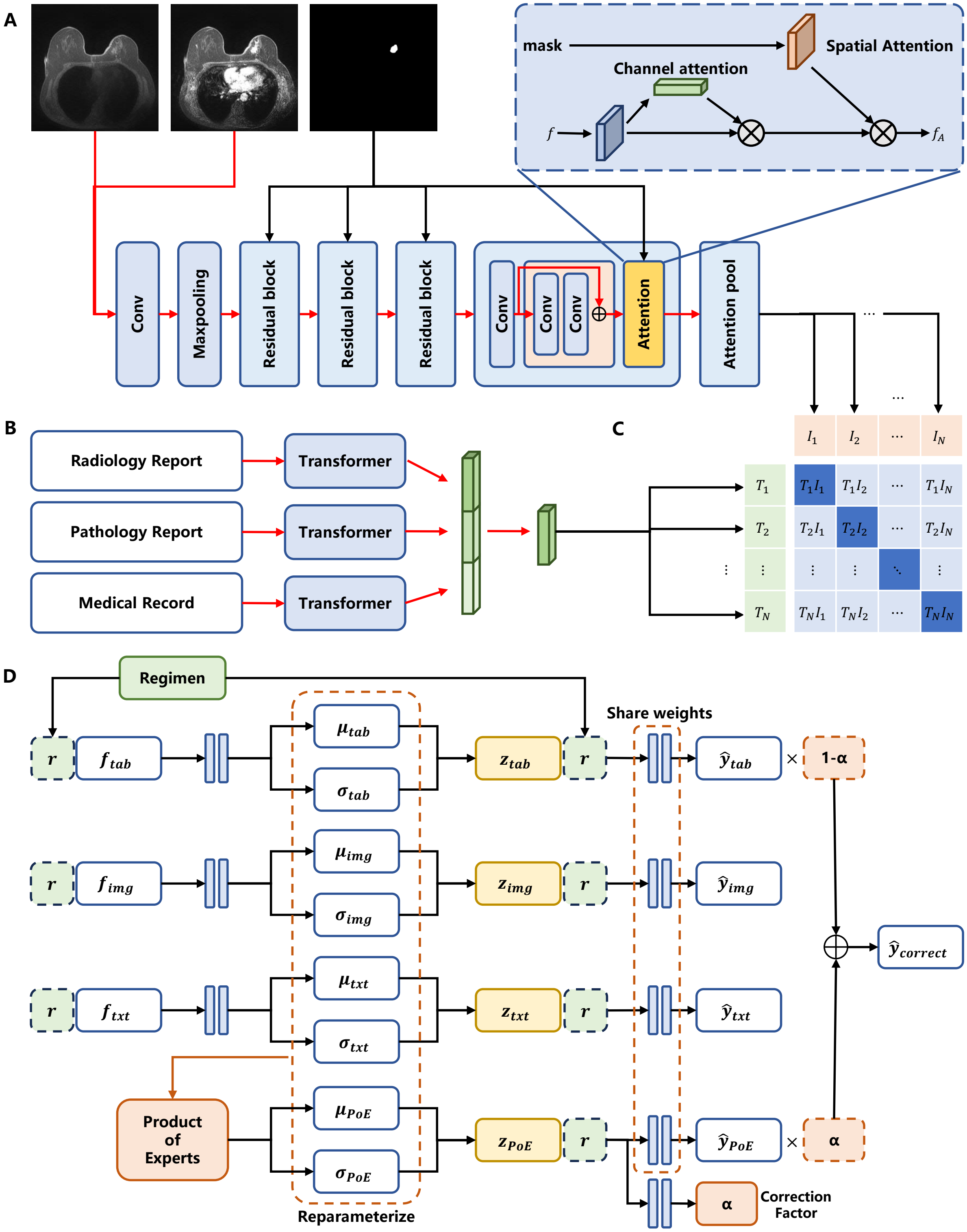

Figure S3. Overview of the model architecture. (A) Details of the image encoder. (B) Details of the text encoder. (C) CLIP for MRI images and medical reports. (D) Variational distribution combination for clinical data, MRI images, and medical reports.

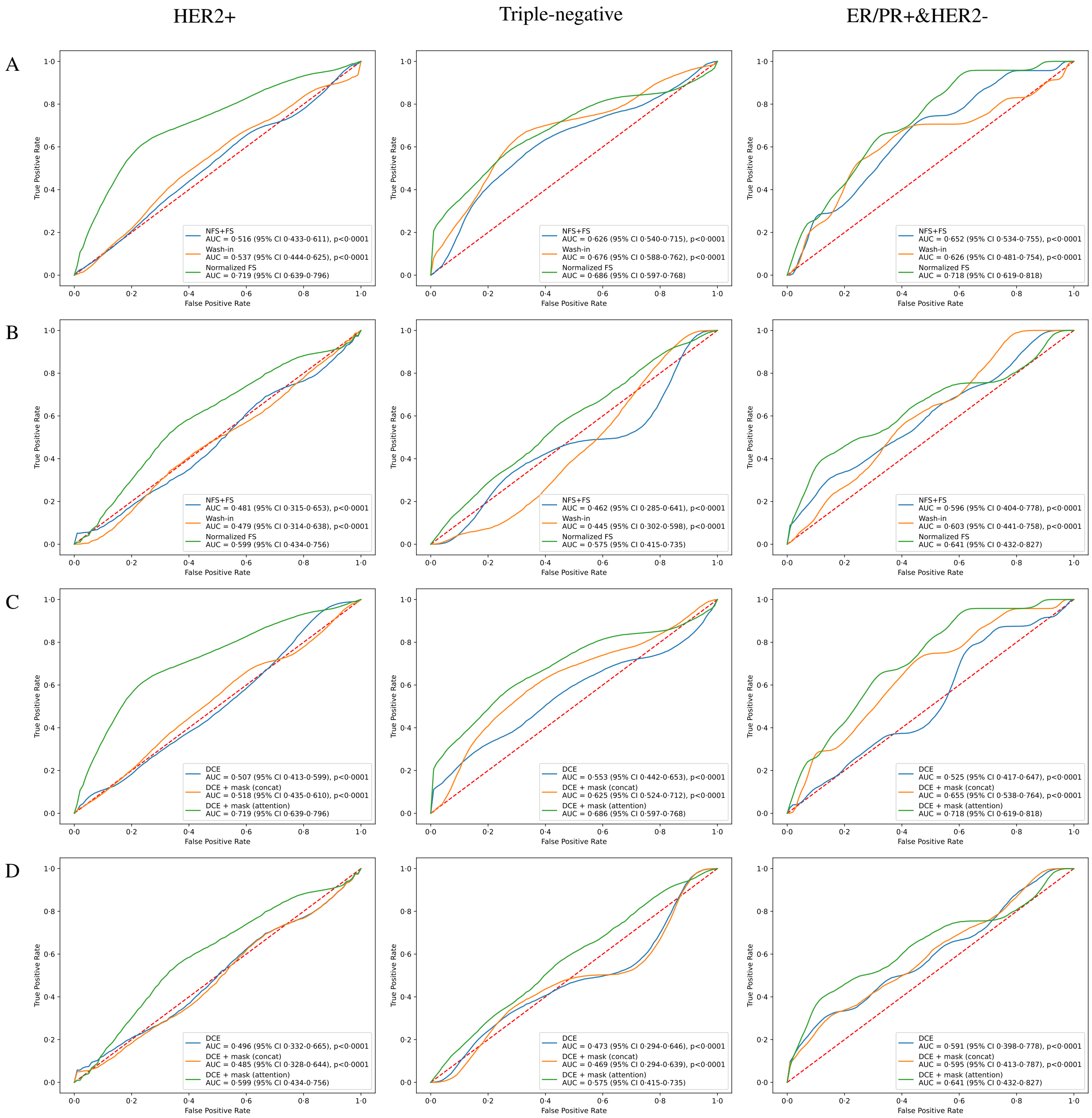

Figure S4. Ablation study of response models for pCR prediction in the NKI-internal validation set and DUKE external validation cohort. Ablation study on using normalized FS images, using a mixture of FS and NFS images, or using wash-in images (A: NKI, B: DUKE). Ablation study on utilizing the segmentation mask as an attention map for the encoder, concatenating the segmentation mask with DCE-MRI, or not using it (C: NKI, D: DUKE).

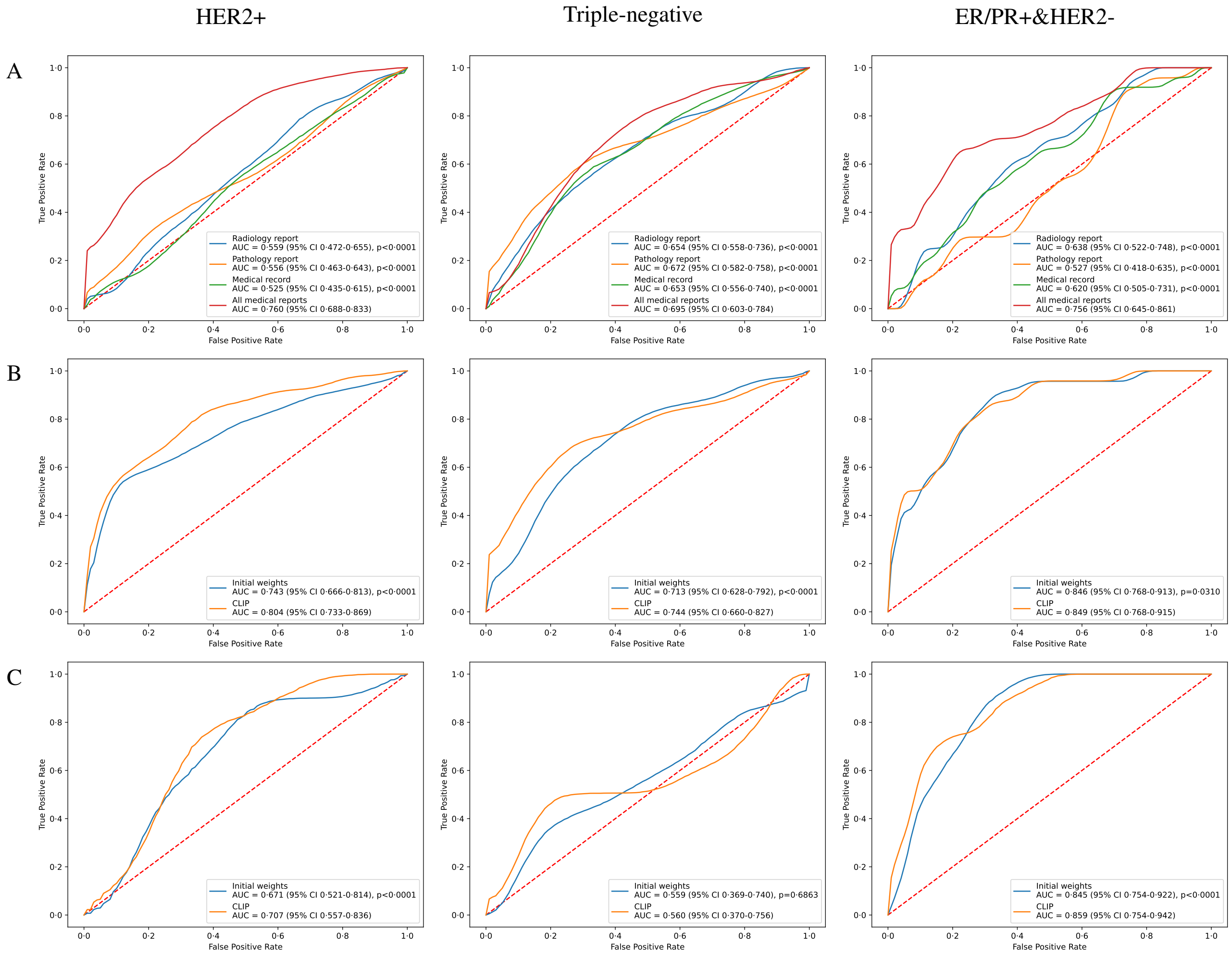

Figure S5. Ablation study of response models for pCR prediction in the NKI-internal validation set and DUKE external validation cohort. Ablation study on using radiology reports, using pathology reports, using medical records, or using all reports (A: NKI). Ablation study on using initial weights or CLIP (B: NKI, C: DUKE).

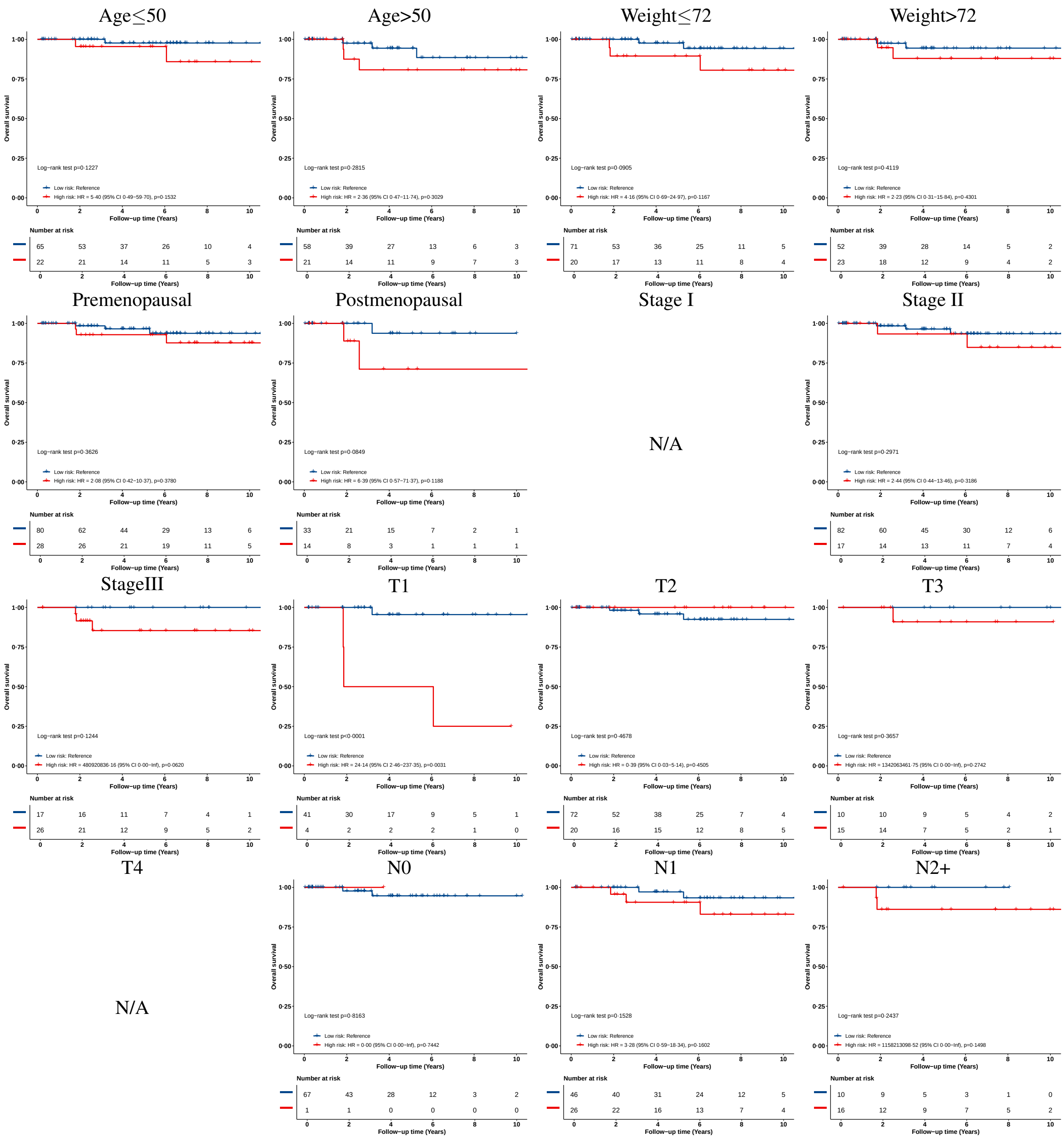

Figure S6. **Kaplan-Meier curves of overall survival in various subgroups of the HER2+ patients in NKI cohort.**

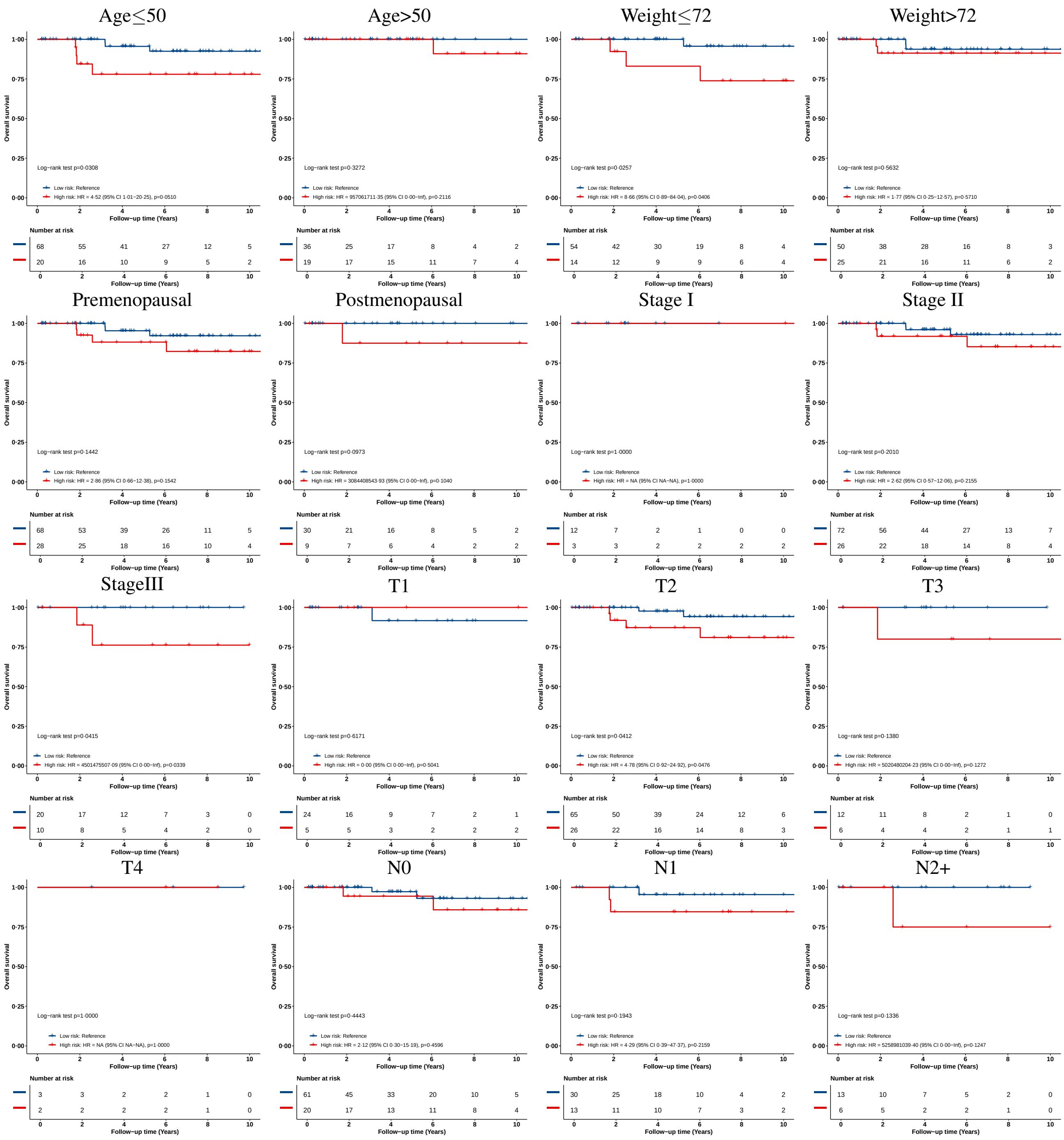

Figure S7. **Kaplan-Meier curves of overall survival in various subgroups of the triple-negative patients in NKI cohort.**

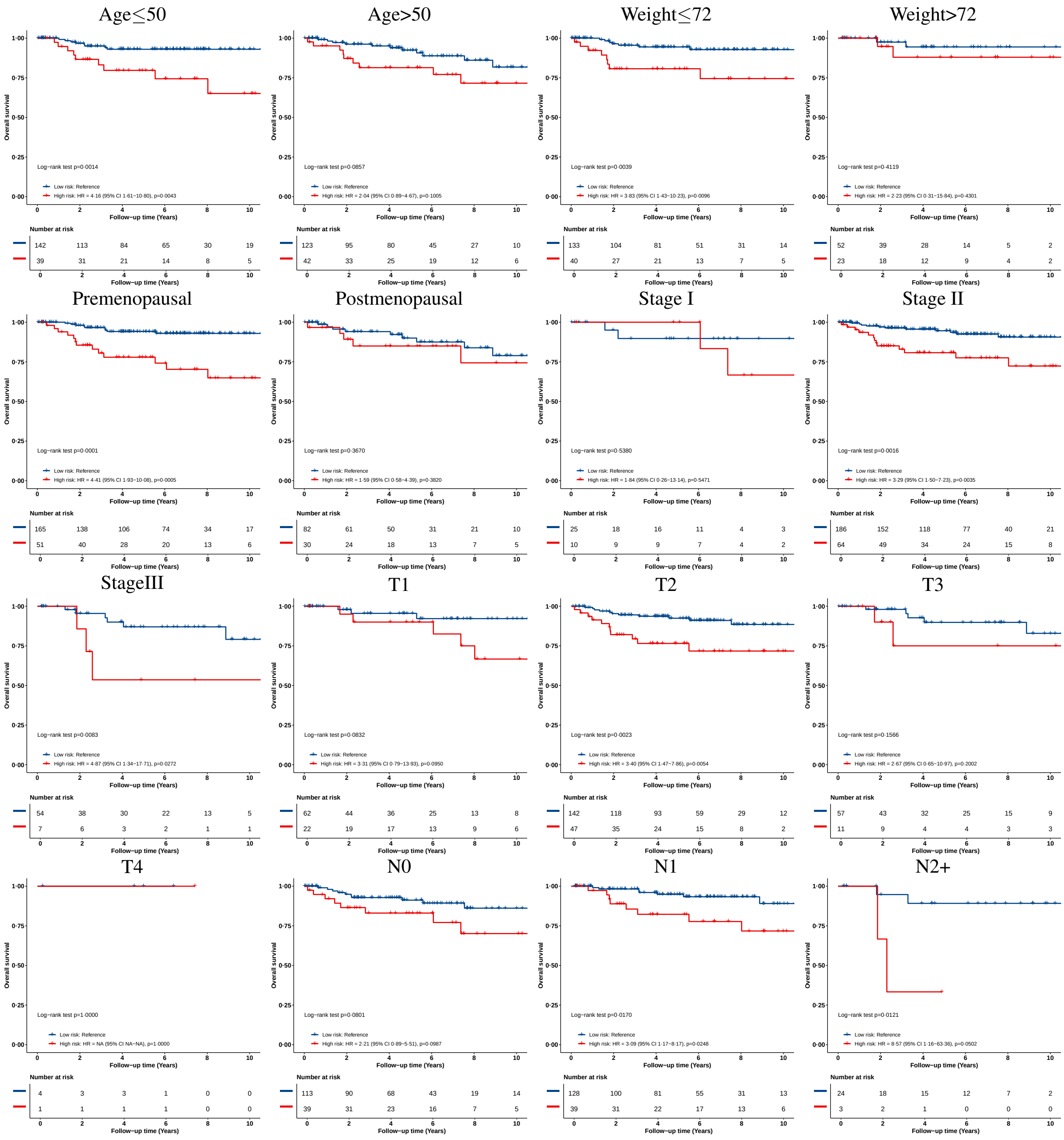

Figure S8. **Kaplan-Meier curves of overall survival in various subgroups of the ER/PR+&HER2- patients in NKI cohort.**

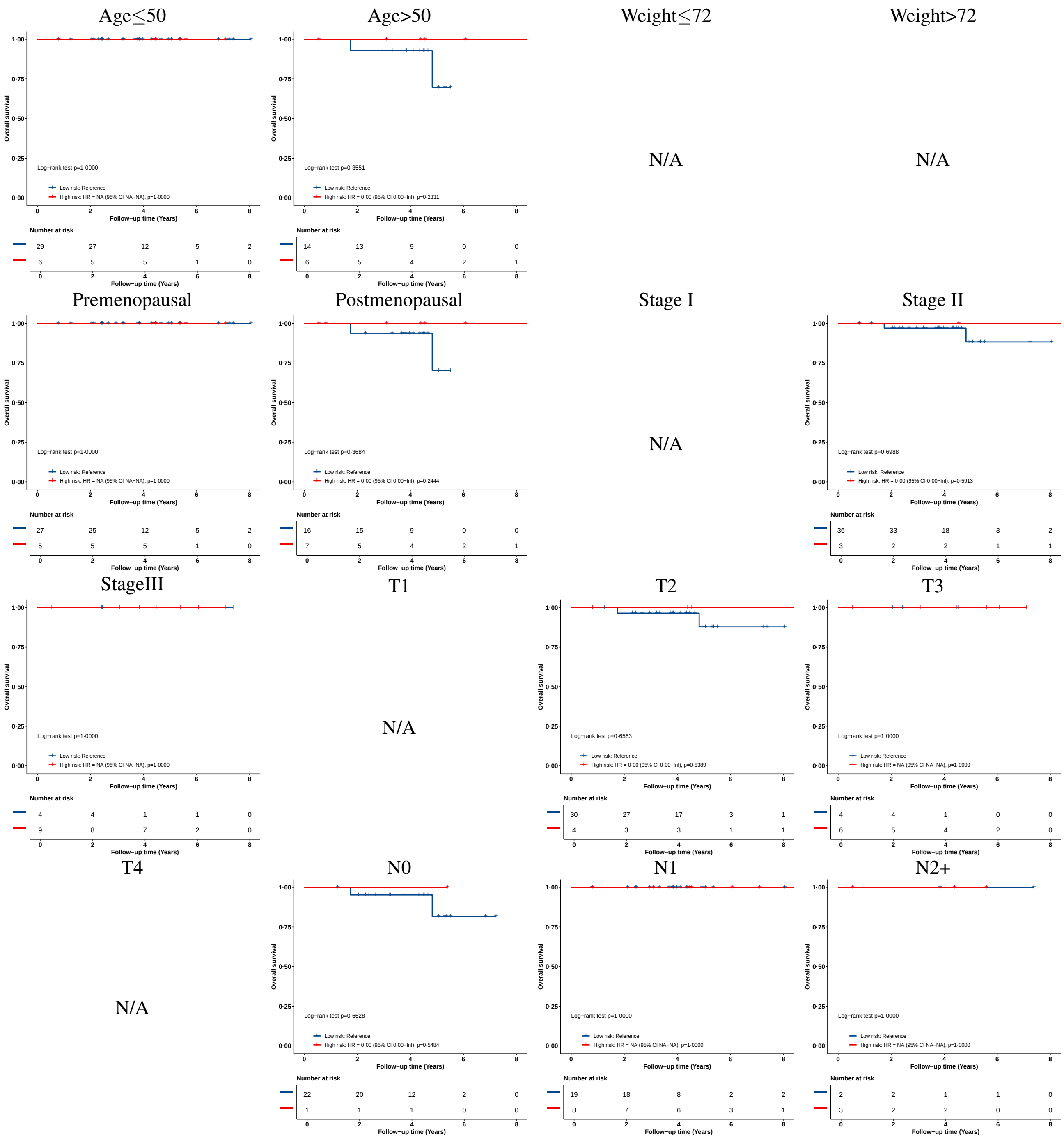

Figure S9. Kaplan-Meier curves of overall survival in various subgroups of the HER2+ patients in DUKE cohort.

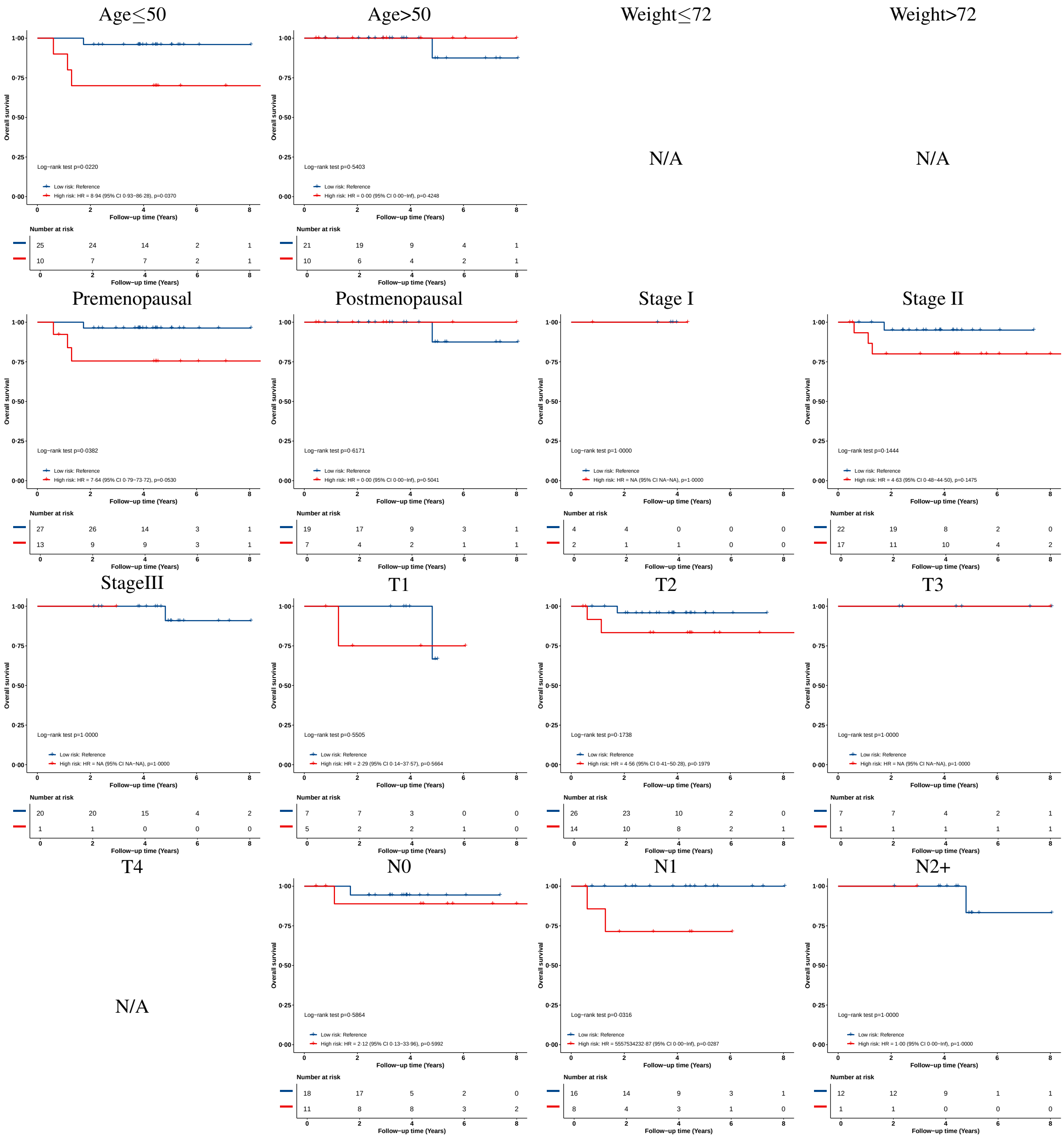

Figure S10. **Kaplan-Meier curves of overall survival in various subgroups of the triple-negative patients in DUKE cohort.**

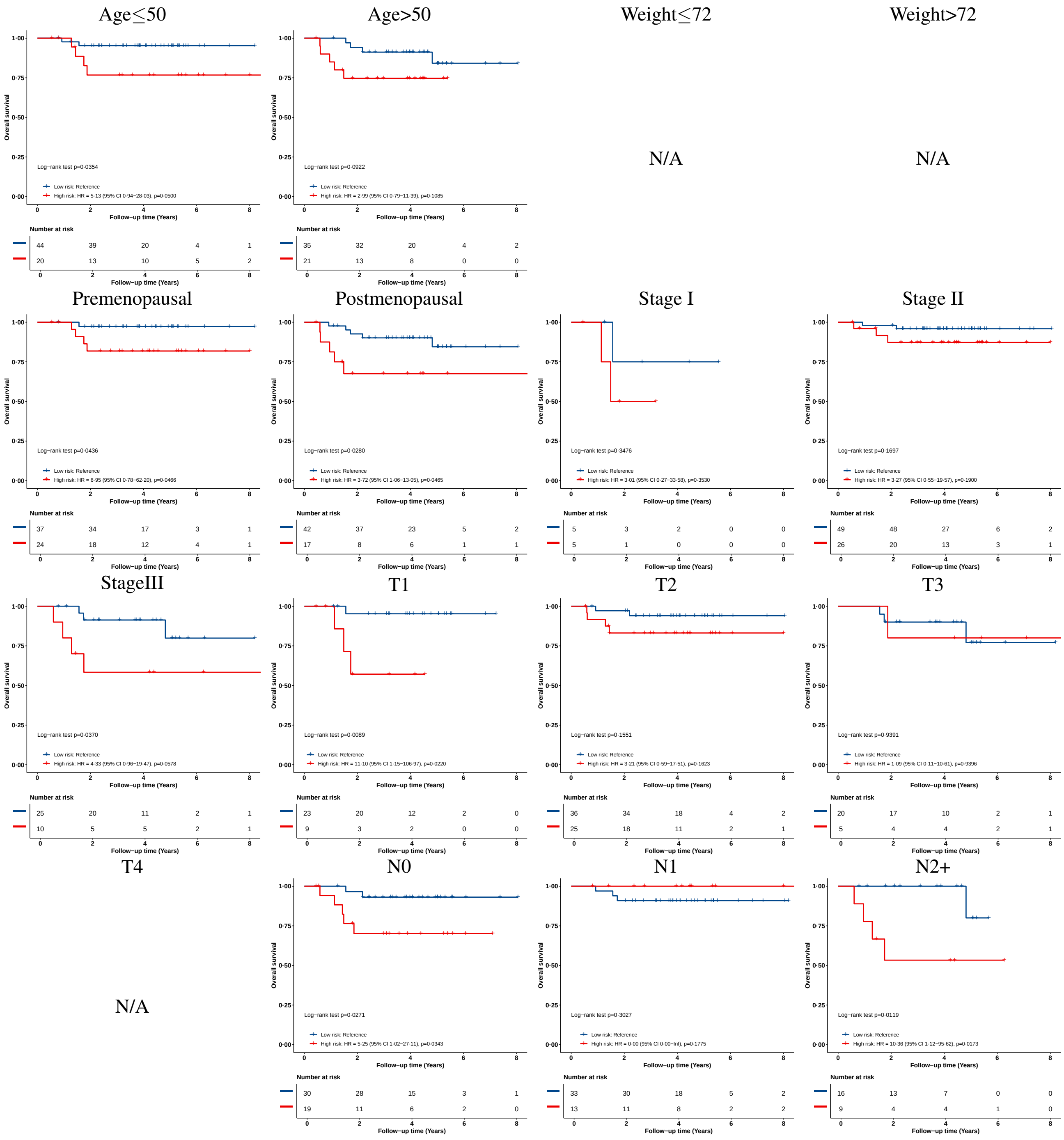

Figure S11. Kaplan-Meier curves of overall survival in various subgroups of the ER/PR+&HER2- patients in DUKE cohort.

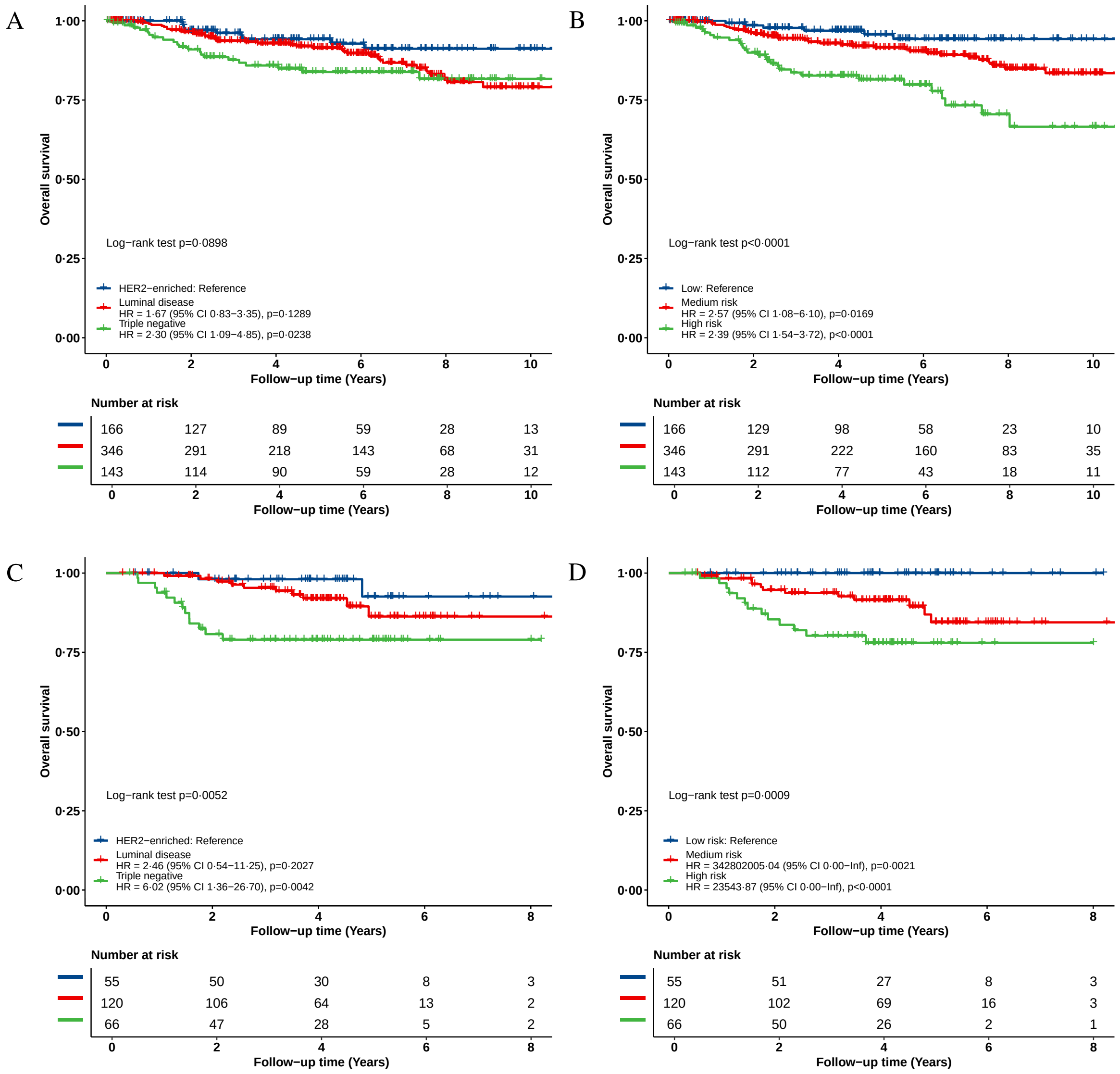

Figure S12. Kaplan-Meier curves of overall survival for comparison of group stratification based on molecular subtype or risk score. (A) for NKI cohort and (C) for DUKE cohort are stratification based on molecular subtypes, and (B) for NKI cohort and (D) for DUKE cohort are stratification based on risk scores with the thresh hold of (-0.733, 0.436) (B) and (-0.471, 0.668) (D), respectively.

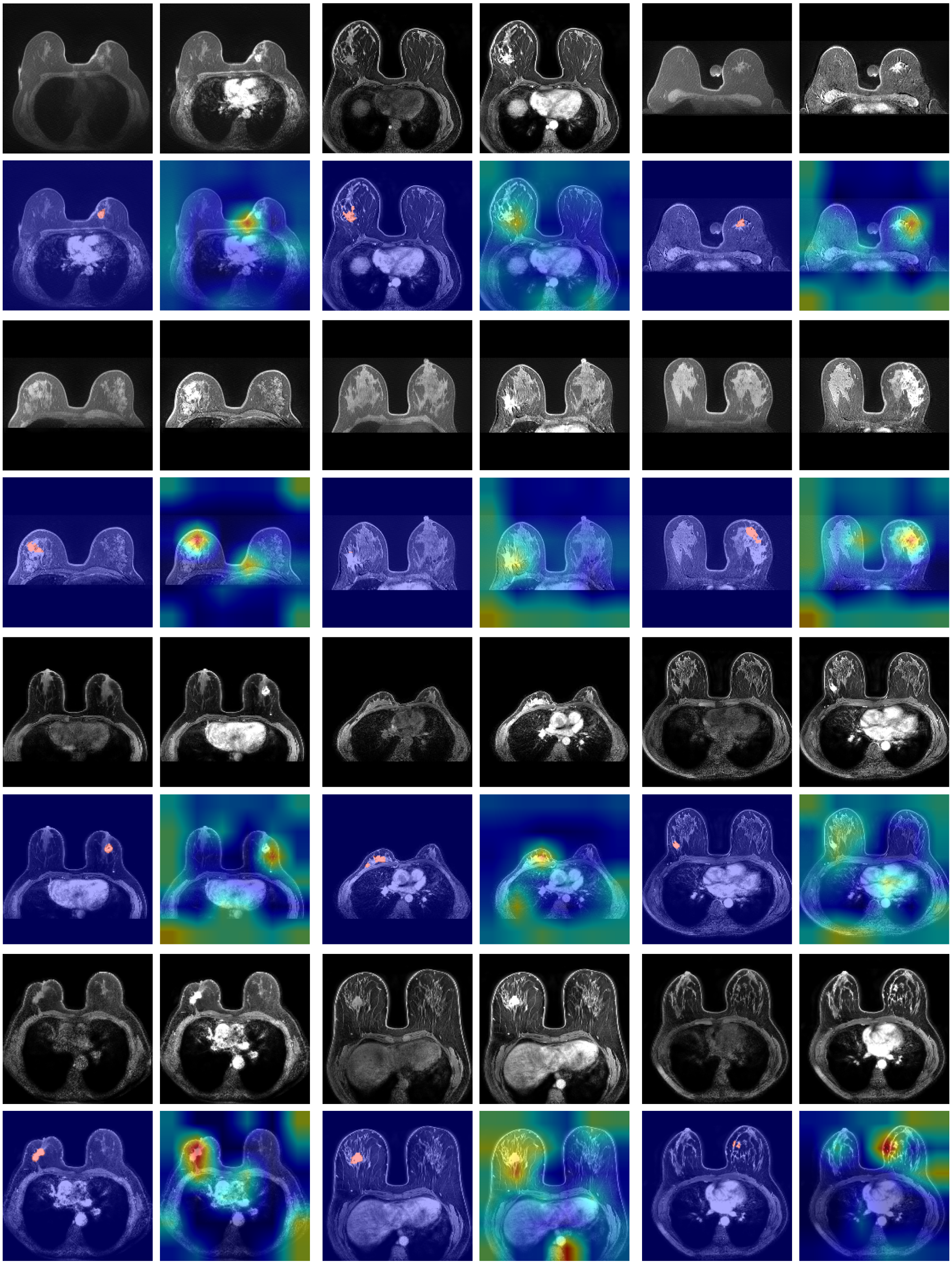

Figure S13. Visualization of interpretability of the image encoder. The figure shows the pre-contrast image (upper left), post-contrast image (upper right), segmentation mask (lower left), and Grad-CAM heatmap (lower right) of each patient.

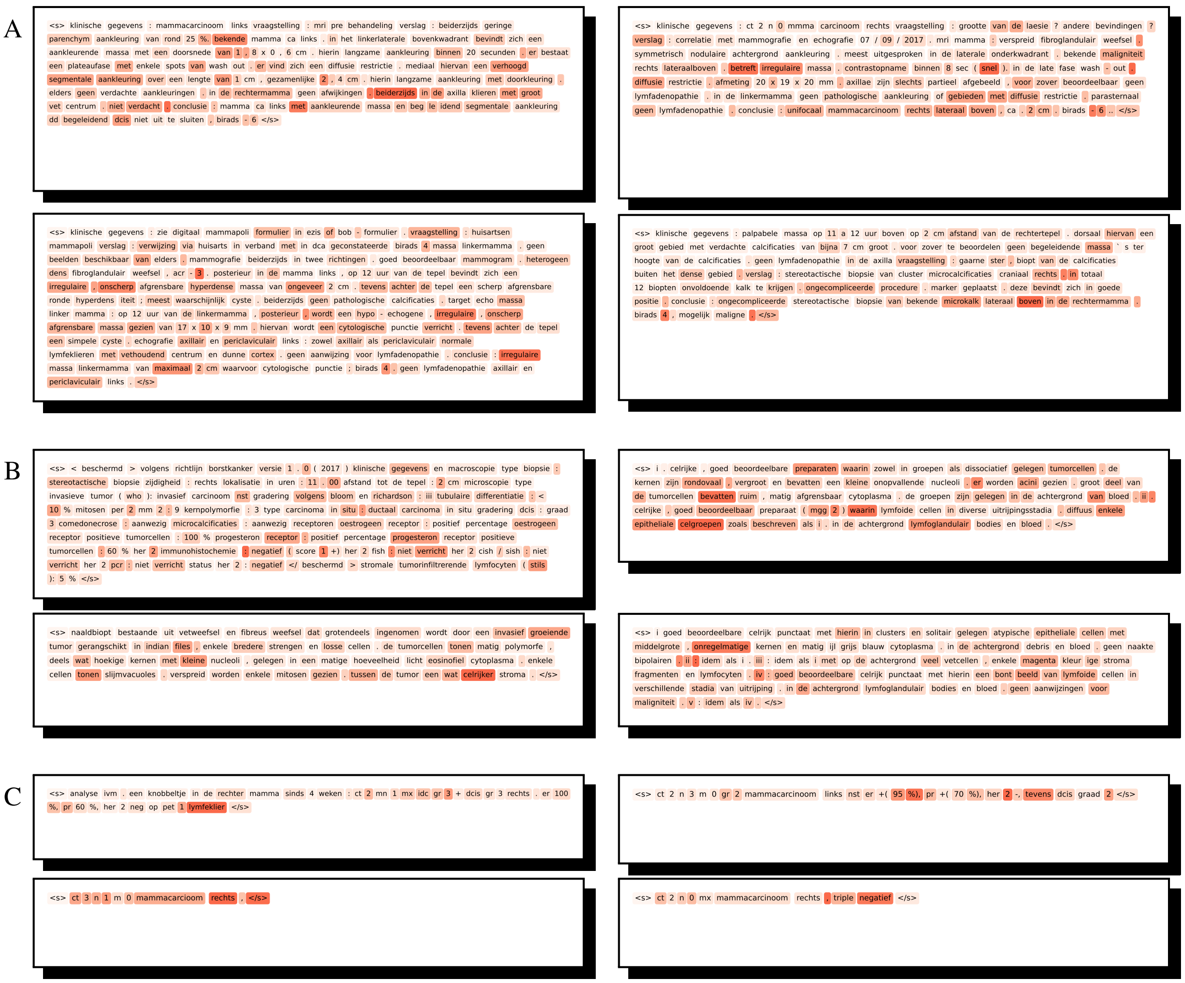

Figure S14. Visualization of correlation heatmaps of the reports. (A): radiology reports, (B): pathology reports, and (C): medical records.

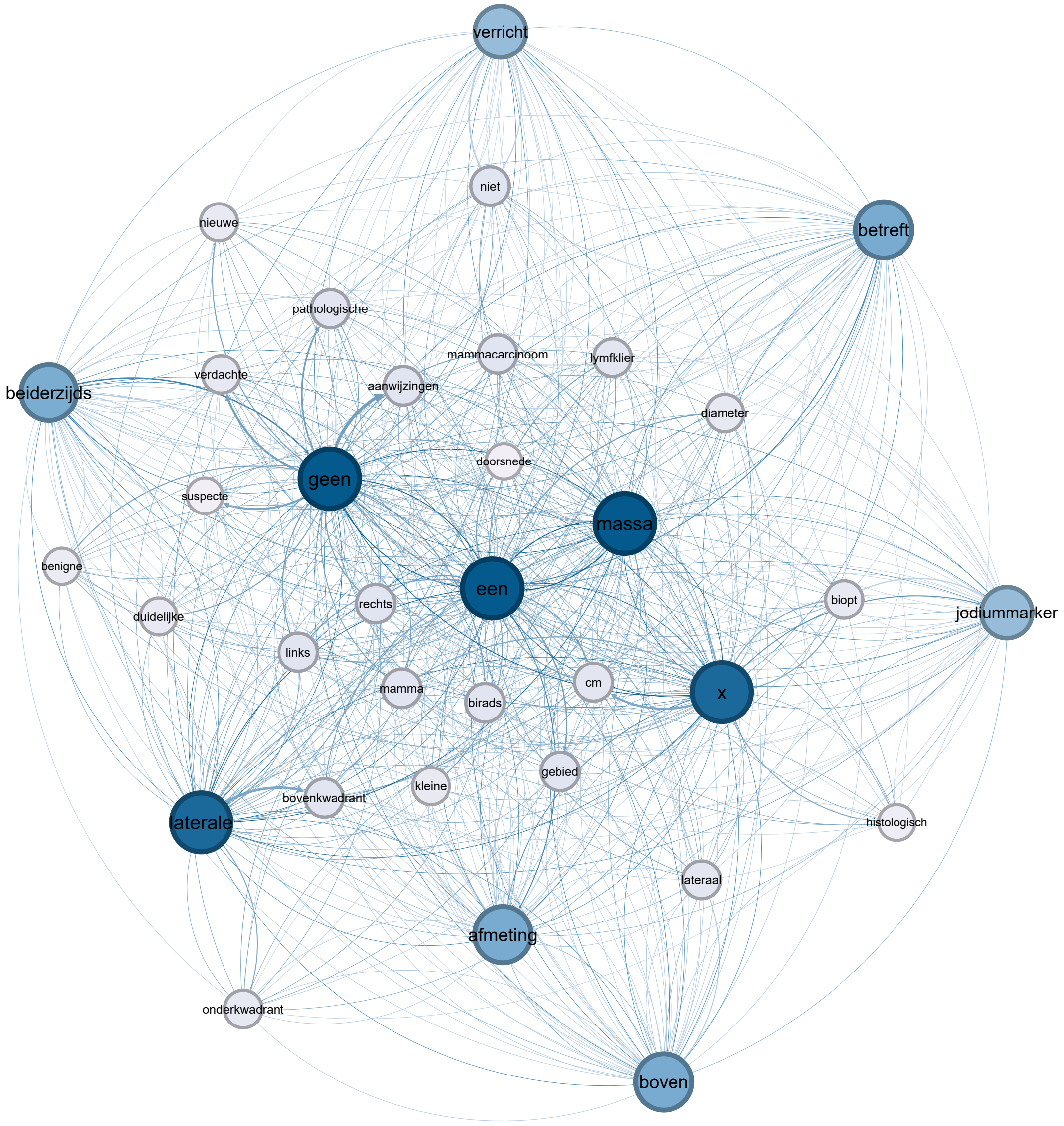

Figure S15. Visualization of word co-occurrence. Translation of Dutch words: aanwijzingen/indications, afmeting/size, beiderzijds/bilateral, benigne/benign, betreft/concerns, biopt/biopsy, birads/BI-RADS, boven/upper, bovenkwadrant/upper quadrant, cm/cm, diameter/diameter, doorsnede/cross section, duidelijke/clear, een/one, gebied/area, geen/no, histologisch/histological, jodiummarker/iodine marker, kleine/small, lateraal/lateral, laterale/lateral, links/left, lymfklier/lymph node, mamma/breast, mammacarcinoom/breast carcinoma, massa/mass, niet/not, nieuwe/new, onderkwadrant/lower quadrant, pathologische/pathological, rechts/right, suspecte/suspect, verdachte/suspicious, verricht/performed, x/X-ray.

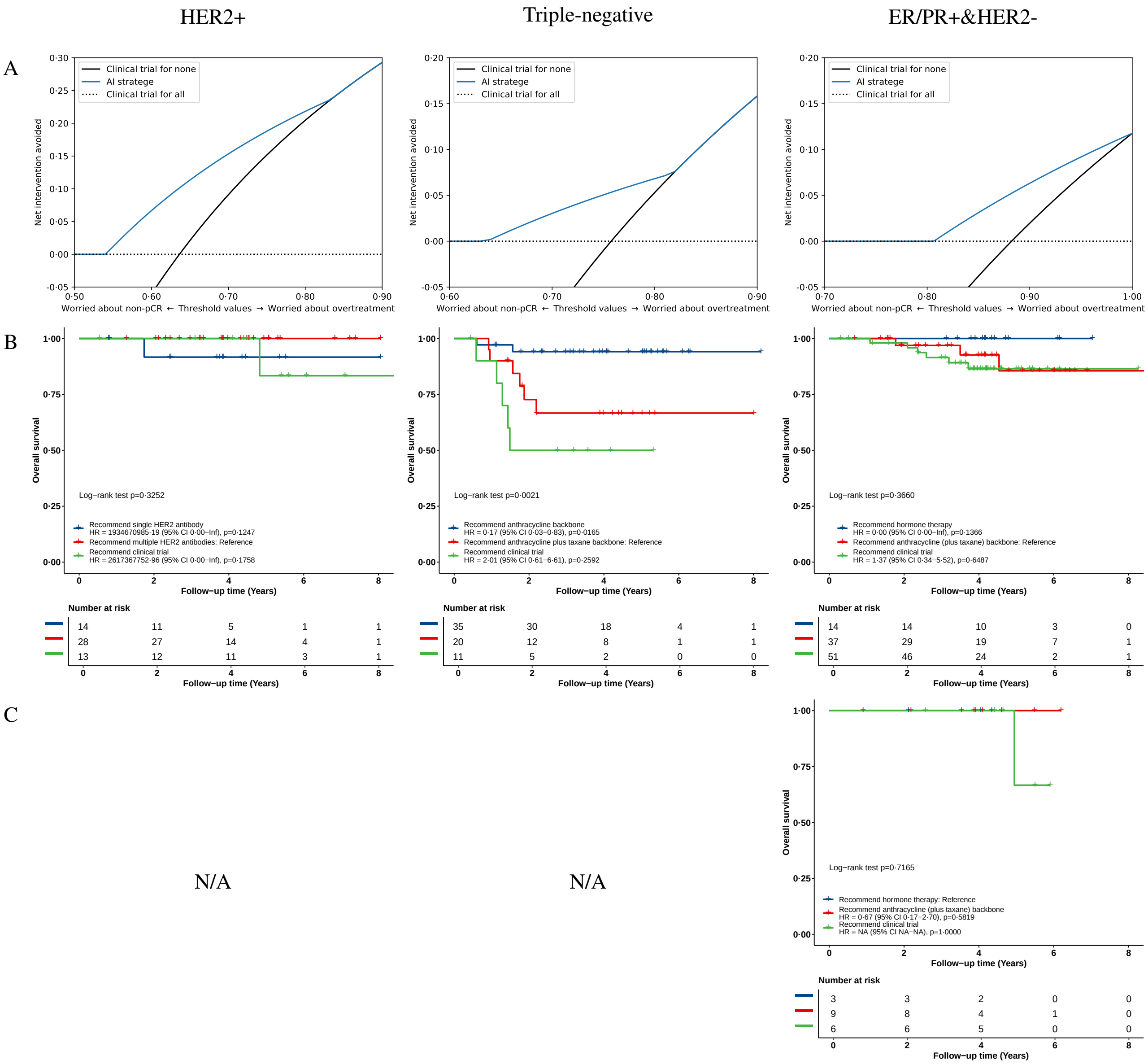

Figure S16. Benefits of AI recommendations in different factual regimen subgroups of patients in the DUKE validation set. Decision curve for AI recommendation on patients with factual regimens of higher-toxicity regimens (A). Kaplan-Meier survival curve for patients applying model-based three-category regimen recommendation on patients with factual regimens of higher-toxicity regimens (B) and patients with factual regimens of lower-toxicity regimens (C).

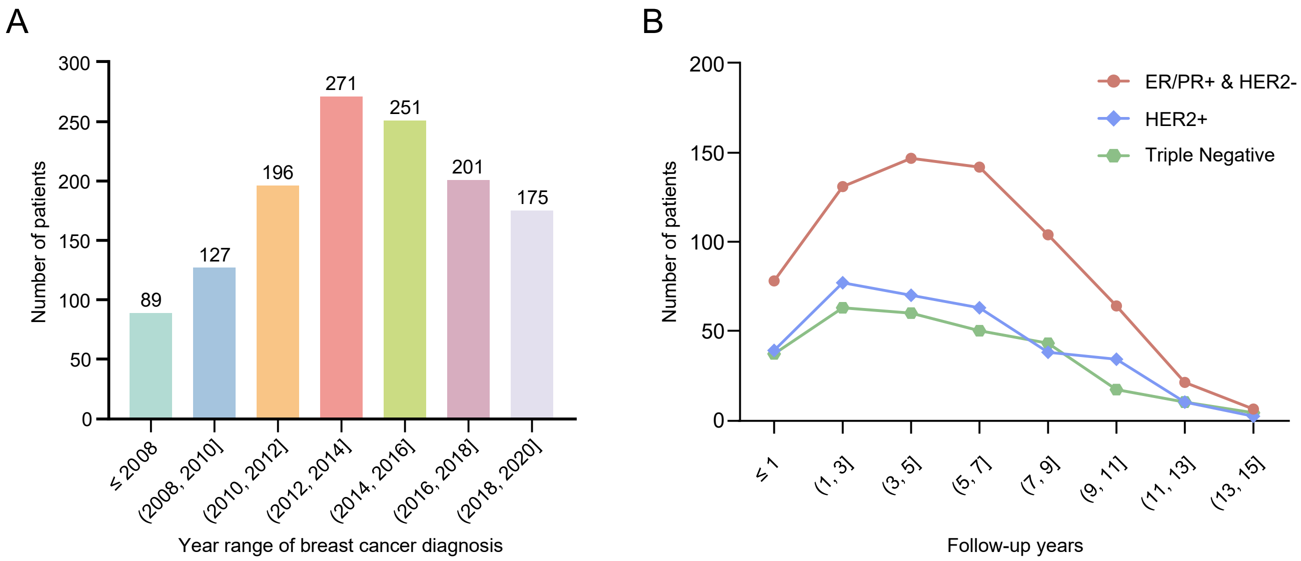

Figure S17. (A) Yearly distribution chart of the number of breast cancer patients diagnosed. (B) Follow-up distribution chart of the number of breast cancer patients.

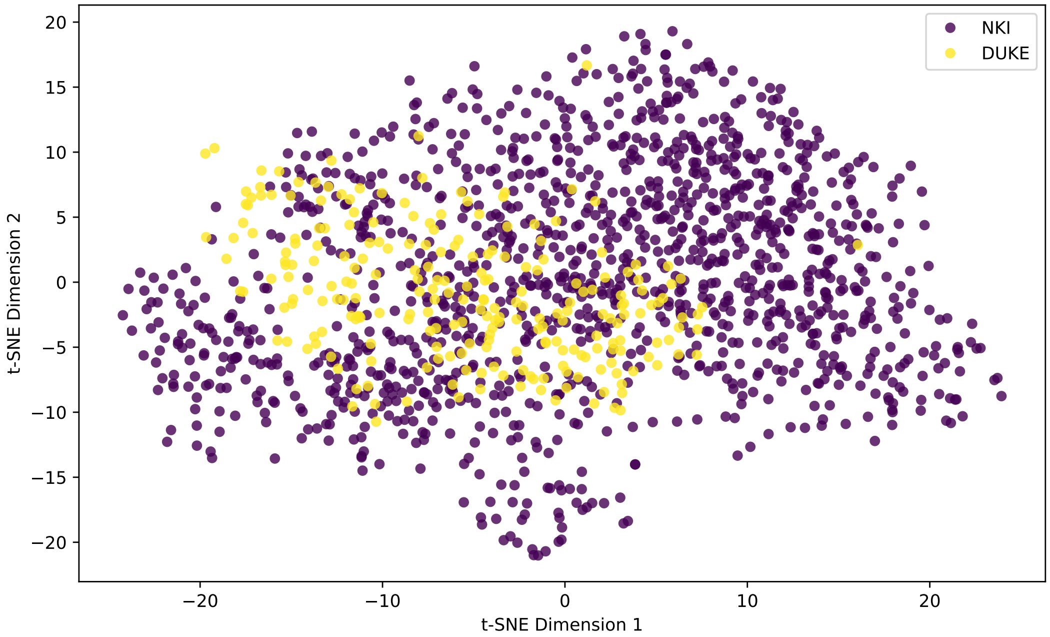

Figure S18. t-SNE of image features between NKI and DUKE cohorts.

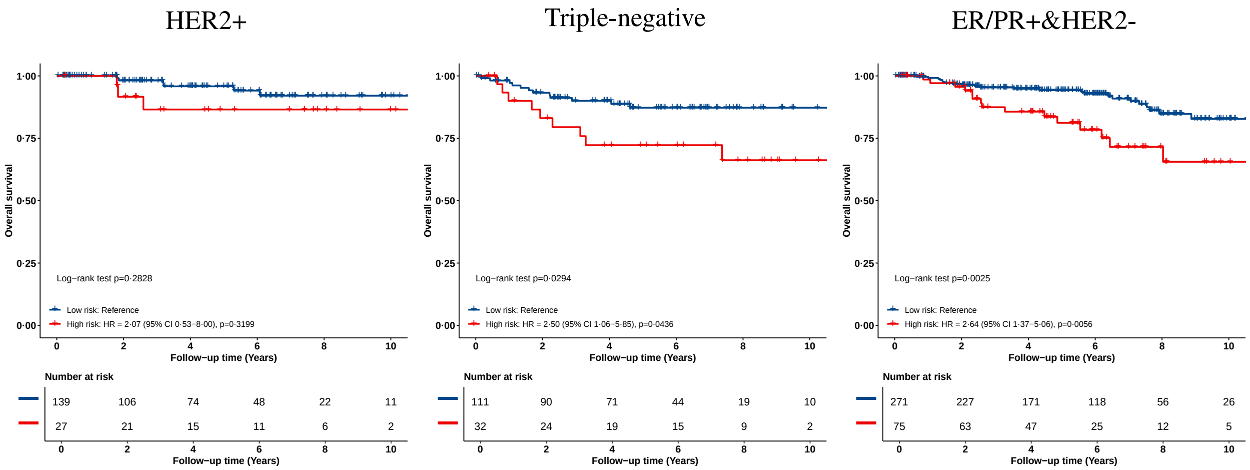

Figure S19. **Kaplan-Meier curves for patients in the NKI cohort stratified into low-risk and high-risk groups based on risk scores predicted by Cox proportional hazards with regularization.**

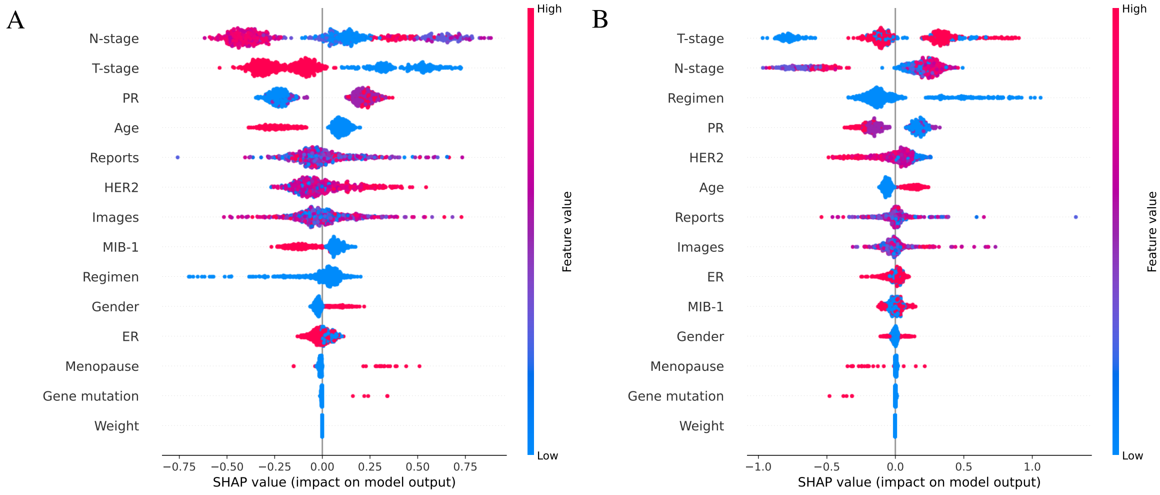

Figure S20. **Feature impact based on SHAP-values in predicting pCR (A) and survival (B).**
